# Messenger RNA-Based Therapeutics and Vaccines: What’s beyond COVID-19?

**DOI:** 10.1021/acsptsci.3c00047

**Published:** 2023-07-03

**Authors:** Dongqiao Li, Cynthia Liu, Yingzhu Li, Rumiana Tenchov, Janet M. Sasso, Di Zhang, Dan Li, Lixue Zou, Xuezhao Wang, Qiongqiong Zhou

**Affiliations:** ‡ Information Center, National Science Library, Chinese Academy of Science, Haidan District, Beijing 100190, P.R. China; § CAS, a division of the American Chemical Society 2540 Olentangy River Rd, Columbus, Ohio 43202, United States

**Keywords:** mRNA, vaccine, therapeutic, COVID-19, infectious disease, cancer

## Abstract

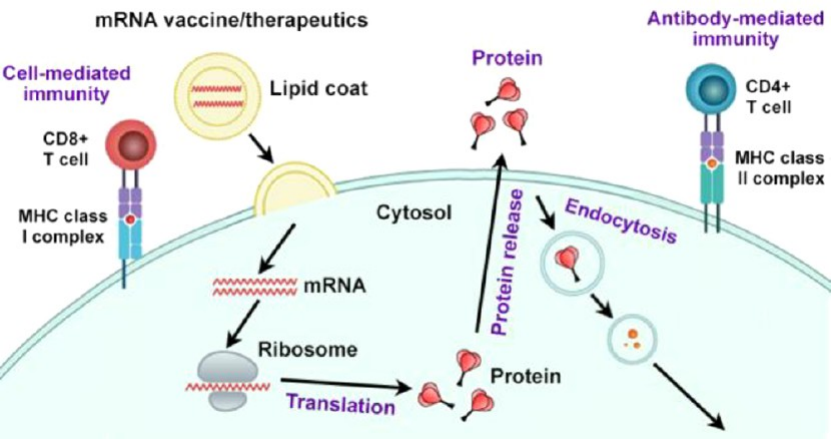

With the rapid success in the development of mRNA vaccines against COVID-19 and with a number of mRNA-based drugs ahead in the pipelines, mRNA has catapulted to the forefront of drug research, demonstrating its substantial effectiveness against a broad range of diseases. As the recent global pandemic gradually fades, we cannot stop thinking about what the world has gained: the realization and validation of the power of mRNA in modern medicine. A significant amount of research has now been concentrated on developing mRNA drugs and vaccine platforms against infectious and immune diseases, cancer, and other debilitating diseases and has demonstrated encouraging results. Here, based on the CAS Content Collection, we provide a landscape view of the current state, outline trends in the research and development of mRNA therapeutics and vaccines, and highlight some notable patents focusing on mRNA therapeutics, vaccines, and delivery systems. Analysis of diseases disclosed in patents also reveals highly investigated diseases for treatments with these medicines. Finally, we provide information about mRNA therapeutics and vaccines in clinical trials. We hope this Review will be useful for understanding the current knowledge in the field of mRNA medicines and will assist in efforts to solve its remaining challenges and revolutionize the treatment of human diseases.

The COVID-19 mRNA vaccines were developed and approved at unprecedented speed and have demonstrated significant effectiveness against infections and acute COVID in the real world. Although the idea of using mRNA as a simple and promising way to deliver vaccines or therapeutic drugs had been around for decades before the onset of the recent global pandemic, the success of mRNA vaccines against COVID has created huge enthusiasm around this concept and significantly boosted development and applications of this class of medicines in other areas. As the pandemic gradually fades, we cannot stop thinking about what the world has gained in this chaos: the realization and validation of the power of mRNA in modern medicine.

Messenger RNA (mRNA) is the molecule that carries genetic information from DNA in the cell nucleus to the cytosol for synthesizing proteins by ribosomes. While most of conventional therapies work by binding and inhibiting hyperactive disease-causing proteins, mRNA therapies can restore protein activities for treating diseases caused by the loss of certain protein functions. Moreover, mRNA therapy is explicit, as defined by the nucleic acid sequence, and very unlikely to have an off-target effect. Compared to antibody or cell therapies, mRNA is also much easier to synthesize and purify on large scales. Another advantage is that mRNA is transient and does not enter the cell nucleus; therefore, it is very unlikely to cause any genetic mutations in cells.

Many key research findings have contributed to the advancement of mRNA’s medical applications. Early research on mRNA’s stability and translational activity provided the foundation for developing mRNA-based vaccines and drugs. Comprehensive exploration of nucleic acids in the 1950s and 1960s brought the discovery of mRNA.^1−3^ Since then, mRNA has been the subject of systematic basic and applied research aimed at various diseases (Figure 1).

**Figure 1:**
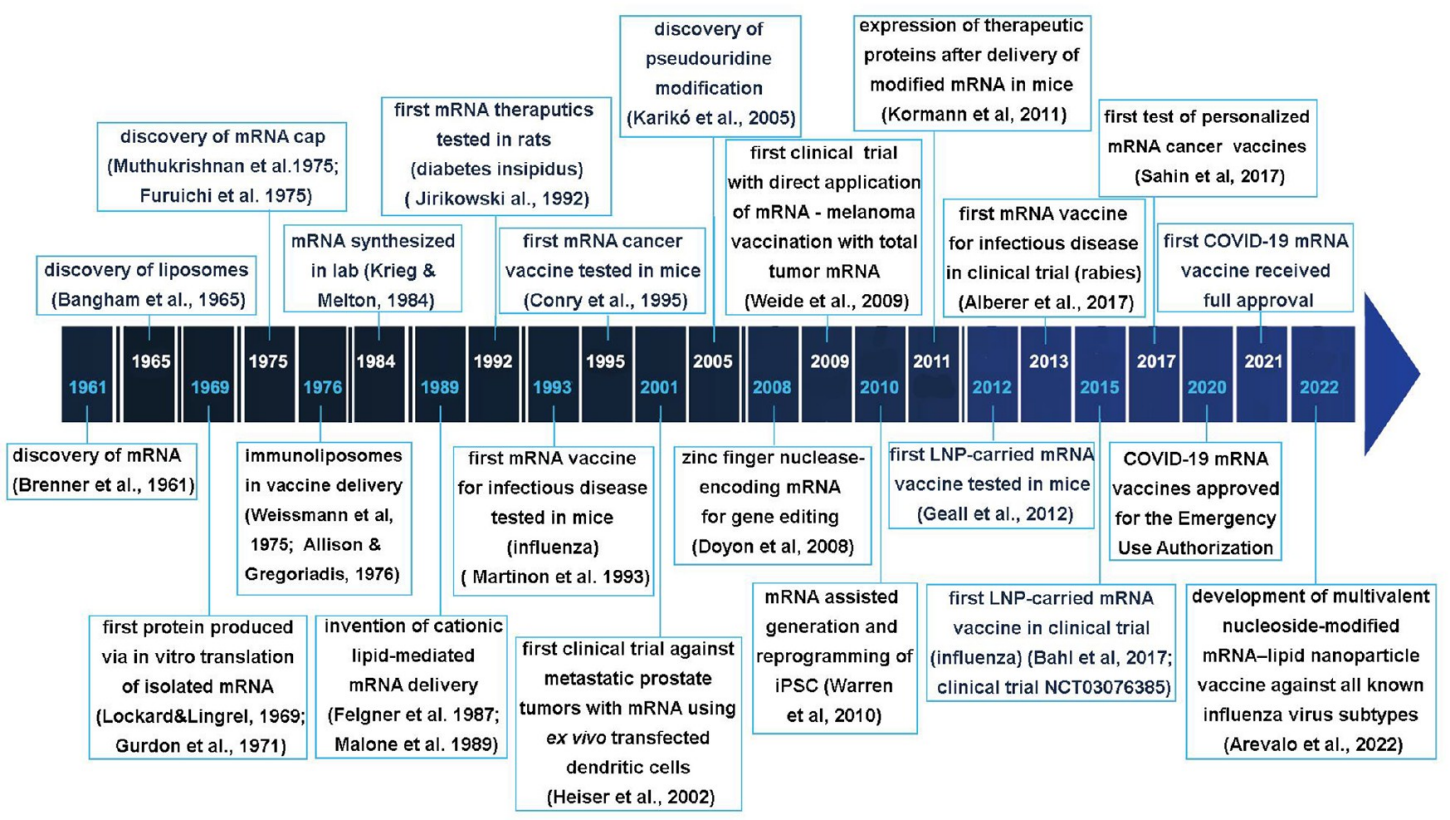
A timeline of the milestone discoveries and key technologies leading to the successful development of mRNA therapeutics and vaccines; work from references (1, 4, 6, 9−13, 20−22, 24, 25, 28−45).

In the first decades after its discovery, the research was mainly focused on understanding the structural and functional aspects of mRNA and its metabolism in eukaryotic cells, in parallel with developing tools for mRNA recombinant engineering. Later, the 5′-cap on mRNA was discovered.^4,5^ In the 1980s, *in vitro* transcription from engineered DNA templates by means of a bacteriophage SP6 promoter and RNA polymerase led to the production of mRNA in cell-free systems.^6^ Although attempts in using liposomes to deliver mRNA into cells to induce protein expression date from the 1970s,^7,8^ the invention of cationic lipids^9^ was the decisive step in enabling nucleic acid transport into cells which resulted in the first cationic lipid-assisted mRNA delivery.^9,10^

In the 1990s, preclinical evaluation of in vitro mRNA transcription began for applications such as protein substitution and cancer and infectious diseases vaccinations.^11−19^ In 1992, a team of scientists working at Scripps Research Institute used mRNA to transiently reverse diabetes insipidus in rats.^11^ Albeit the concept of mRNA vaccines sounds relatively new, it was actually first suggested in 1995, for encoding cancer antigens.^13^ The accrued expertise was valuable in solving some of the obstacles associated with mRNA pharmaceuticals such as its short half-life and unfavorable immunogenicity.

In 2005, a solution was found on how to prevent activation of the immune response against the injected mRNA per se by inserting a naturally occurring modified nucleoside: pseudouridine.^20^ The invention of the pseudouridine modification and further exploration on mRNA led to the first human trial of a mRNA vaccine against melanoma in 2008.^21^ In the following years, numerous preclinical and clinical trials on mRNA-based vaccines against infectious diseases and cancer were completed.^22,23^ In 2009, the first trial on cancer immunotherapy using mRNA-based vaccines in human subjects with metastatic melanoma was conducted.^21^ In 2010, it was shown that pseudouridine-modified mRNA might be applied as a safe approach for effectively reprogramming cells to pluripotency.^24^ The first clinical trial of personalized mRNA-based cancer vaccine was performed in 2017.^25^ In 2021, a successful use of lipid nanoparticles (LNPs) comprising *Streptococcus pyogenes* Cas9 mRNA and a CRISPR guide RNA in patients with transthyretin amyloidosis with polyneuropathy was reported.^26^

Two human mRNA vaccines against COVID-19 received Emergency Use Authorization in 2020 and were finally approved in 2021.^27−29^ This was only brought about by decades of research on mRNA-based therapeutics. The lessons learned during the COVID-19 mRNA vaccines development were recently applied in formulating a multivalent nucleoside-modified mRNA flu vaccine.^30^

As it became clear that mRNA vaccines provide a promising alternative to conventional vaccine approaches due to their high efficiency, potential for rapid development, low-cost manufacture, and capacity for scale-up, as well as safe administration, significant efforts have been concentrated on developing mRNA drug and vaccine platforms against infectious diseases, cancer, and other debilitating diseases and have demonstrated encouraging results.

This Review provides a detailed overview of mRNA drugs and vaccines and considers future directions and challenges in advancing this promising platform to widespread therapeutic use. We examine data from the CAS Content Collection,^46^ the largest human-curated collection of published scientific information and analyze the publication landscape of recent research in order to reveal the research trends in published documents and to provide insights into the scientific advances in the area. We also discuss the evolution of the key concepts in the field, the major technologies, and their development pipelines with company research focuses, disease categories, development stages, and publication trends. We hope that this report can serve as a useful resource for understanding the current state of knowledge in the field of mRNA medicines and the remaining challenges to fulfill the potential of this new class of medicines.

## Landscape of Scientific Publications Related to mRNA Therapeutics and Vaccines

### Trend of mRNA Publications over Time

**Figure 2:**
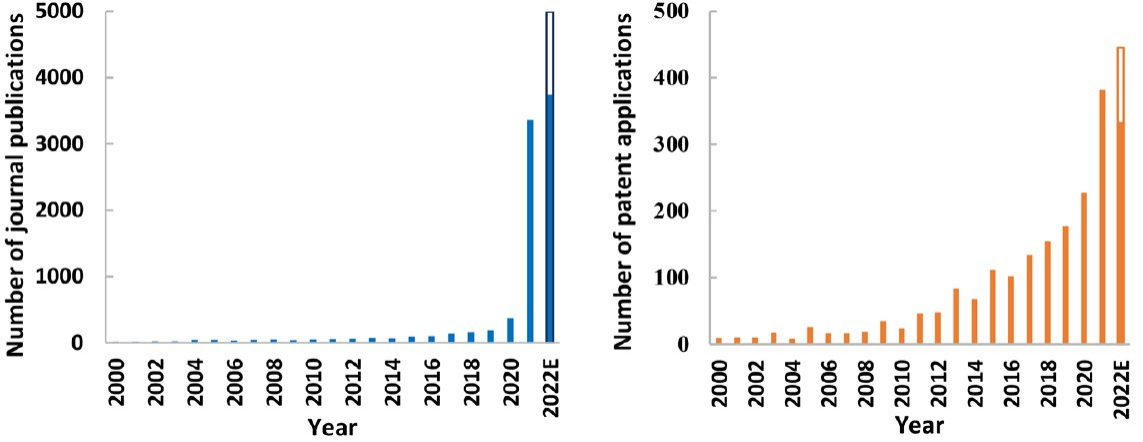
Annual number of published journal articles (left) and patents (right) on mRNA therapeutics and vaccines. The data for 2022 include extrapolated numbers for October to December 2022.

Based on the analysis of CAS document collection, a total of 9,322 research papers have been published in the field of mRNA therapeutics and vaccines. Due to the small number of published papers before 2000, the analysis of the development trend focused on those papers published since 2000. As shown in the left panel of Figure 2, the number of published papers in this area showed a slow growth prior to 2020 followed by a significant increase each year afterward. From 2000 to 2019, the annual number of publications in the global mRNA field was less than 200, and the growth in publication was relatively slow. Due to the impact of the novel coronavirus outbreak at the end of 2019, mRNA technology has attracted wide attention from researchers. After 2020, the number of published papers in this area has shown a rapid growth trend, with the number of published papers increasing to 3,361 in 2021 and nearly 5,000 in 2022.

A total of 2,089 patents related to mRNA therapeutics and vaccines have been published worldwide, according to CAS document collection. Due to the small number of patents before 2000, the trend analysis also focused on the analysis of patents since 2000. As shown in the right panel of Figure 2, the number of patents each year grew slowly from 2000 to 2010 with some fluctuations, and the annual publications were all below 30. Between 2011 and 2019, the number of mRNA-related patents worldwide increased from 46 to 177 each year. Stimulated by the COVID-19 pandemic, the annual number of patents increased dramatically after 2020, increasing to 382 in 2021 and likely to nearly 450 in 2022. We also performed trend analyses for patents on mRNA therapeutic, mRNA vaccines and delivery systems separately. The results for those are described in the Supporting Information.

## Distribution of Research Topics

### Distribution of Topics in Journal Publications

**Figure 3:**
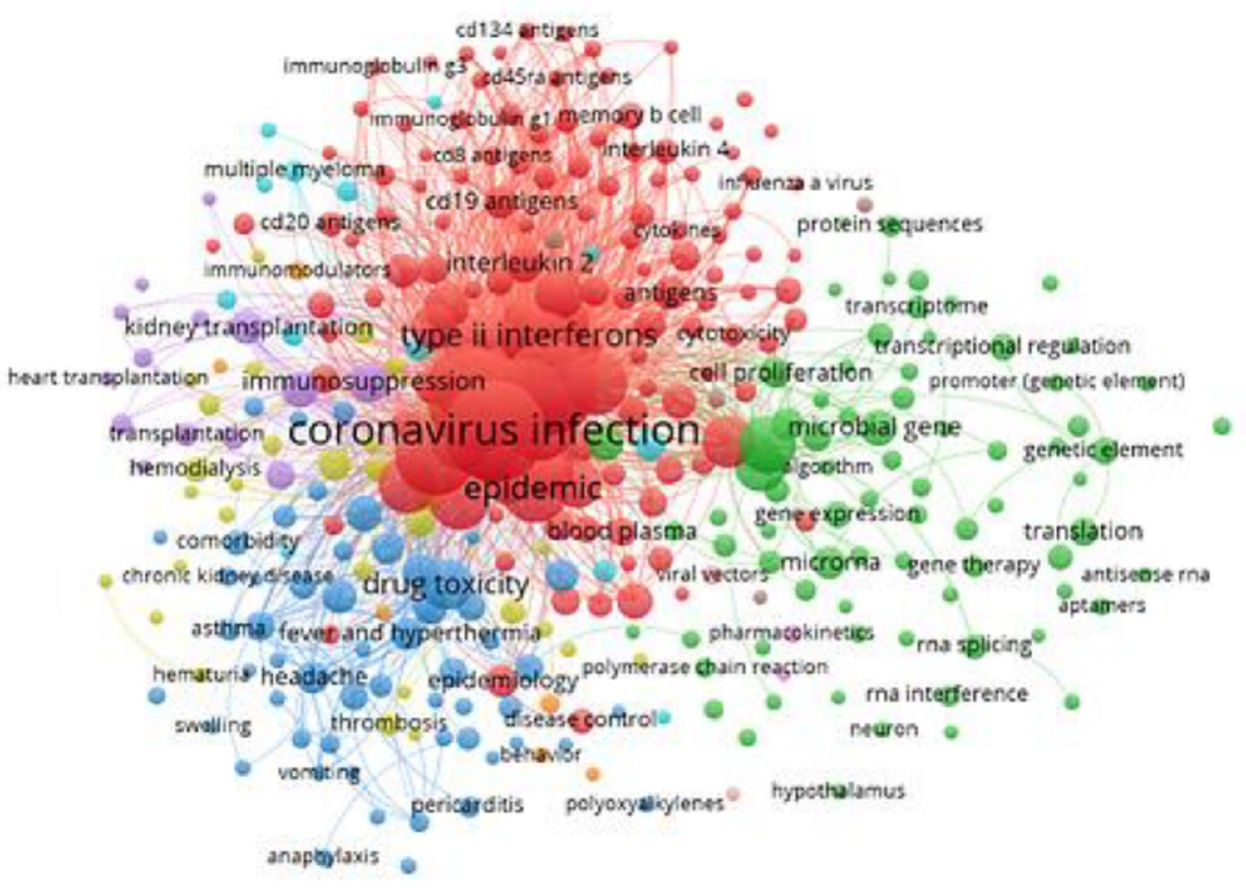
Concepts and their co-occurrence in journal articles related to mRNA therapeutics or vaccines.

This report then examined CAS-indexed concepts in mRNA therapeutic and vaccine journal articles in order to reveal emerging trends or the more specific focus of research and development in this field (Figure 3). From the distribution of research topics based on the index concepts, it seems that the research has thus far focused on research in immunology, mechanisms of action, and disease indications. Among the immune research (red dots), key concepts include immunoglobulin G, viral spike glycoproteins, neutralizing antibodies, immunogenicity, etc. In terms of mechanism of action studies (green dots), key concepts include signal transduction, transcriptional regulation, genetic elements, RNA splicing, etc. In the treatment of diseases (blue dots), key concepts include diabetes, hypertension, myocarditis, and cardiovascular disease. In terms of indicator studies (yellow dots), key concepts include c-reactive protein, leukocyte, blood platelet, etc. Conceivably, due to the outbreak of the COVID pandemic and the development of mRNA vaccines against this disease, the cluster surrounding coronavirus infection accounts for a very large portion of journal publications.

### Distribution of Topics in Patents

**Figure 4:**
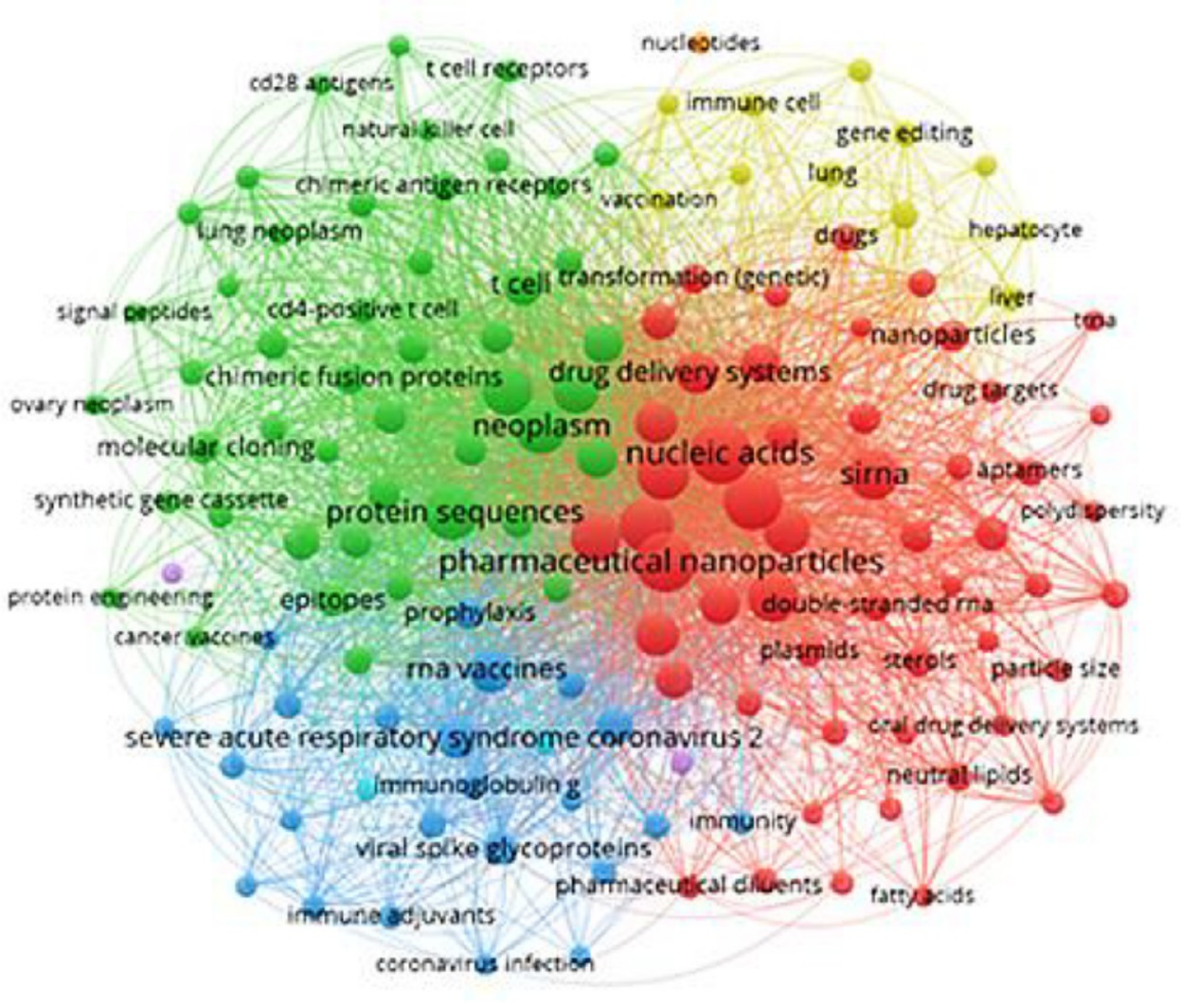
Concepts and their co-occurrence in patents related to mRNA therapeutics or vaccines.

Key concepts in the field of mRNA therapeutics and vaccines were also examined with a concept cluster analysis for patents within the CAS Content Collection (Figure 4). Unlike journals, a significant portion of the patents focused on drug delivery such as nanoparticles (red dots) and immunotherapy in addition to SARS-CoV-2-related studies, etc. In terms of immunotherapy (green dots), key concepts include chimeric fusion proteins, chimeric antigen receptors, cancer immunotherapy, T cell receptors, etc. In other treatment aspects (blue dots), key concepts include RNA vaccines, immune adjuvants, etc. The high occurrence of all these terms reflects the emphasis of most R&D activities in this area.

## Major Countries Contributing to the R&D of mRNA Therapeutics and Vaccines

### Top Countries/Regions

**Figure 5:**
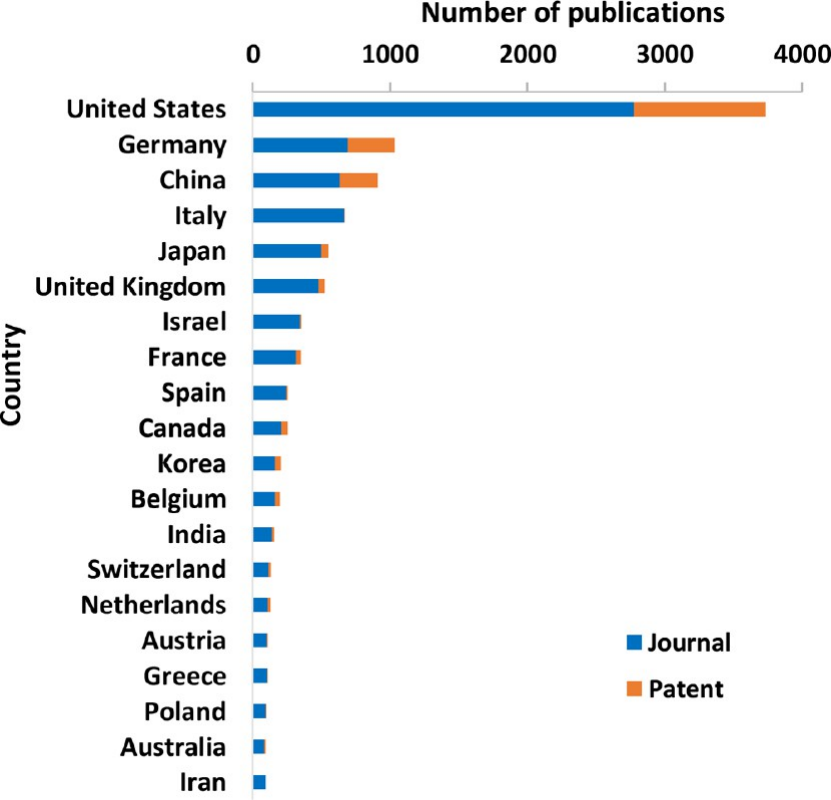
Major countries/regions with journal articles and patents related to mRNA therapeutics or vaccines.

Among the top 20 countries and regions in terms of scientific research output (Figure 5), the United States has the largest scientific research output in both journal articles and patents, with 29.8% and 45.9% of the world’s total output in these two types of publications, respectively, and significantly outnumbers those by other countries/regions. Germany ranks distantly the second in total scientific output, accounting for 7.4% of journal articles and 16.5% of patents. China ranks the third in scientific research output, with 6.8% of journal articles and 13.4% of patents. Italy, Japan, and the United Kingdom ranked fourth, fifth, and sixth, respectively, in total output.

### Patent Output Trend over Time for Top Countries

**Figure 6:**
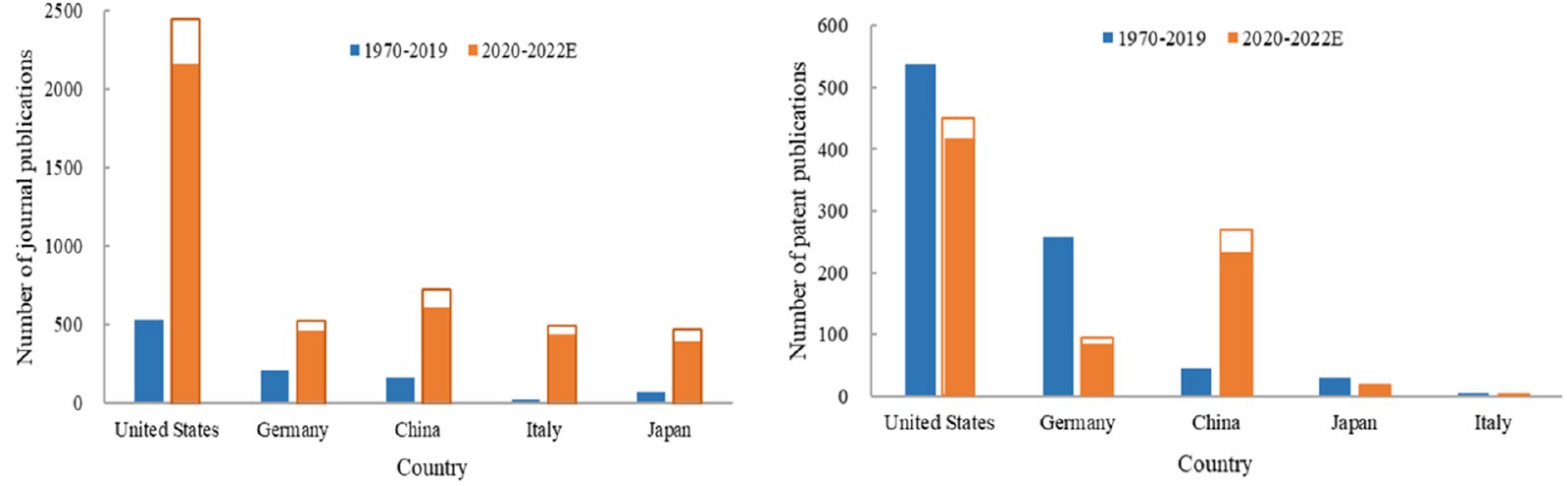
Journal publications (left) and patent output (right) from the top five countries during 1970–2019 vs 2020–2022.

Figure 6 shows the contributions by the top five countries for the periods of 1970–2019 and 2020–2022 (the period following the outbreak of the COVID-19 pandemic). The U.S. is in the lead position in both periods for both journal and patent publications. The number of journal articles published by China from 1970 to 2019 was slightly lower than that of Germany, but increased rapidly during the period of 2020–2022, exceeding Germany during this period. The numbers of journal articles published by Italy and Japan were also small (<80) during 1970–2019 but increased significantly (>390) during 2020–2022 (left panel).

The numbers of patent applications by organizations in the U.S. and Germany during 1970–2019 were higher than those in 2020–2022, indicating the presence of sufficient R&D activities in these two countries before the onset of COVID-19 (Figure 6, right panel). While the numbers of patents from these two countries decreased during the three-year period of 2020–2022, the numbers were still higher than those of other countries, reflecting their leading roles. The trend of patent output from China appears to be different from that of other countries as indicated by the low activity during 1970–2019 and 5-fold increase during the period of 2020–2022, indicating that China is rapidly increasing its technological development in the mRNA therapeutics and vaccines due to the impact of the COVID-19 pandemic.

### Patent Filing Strategies Revealed by Analysis of Patent Application Flows among Major Countries/Regions

**Figure 7:**
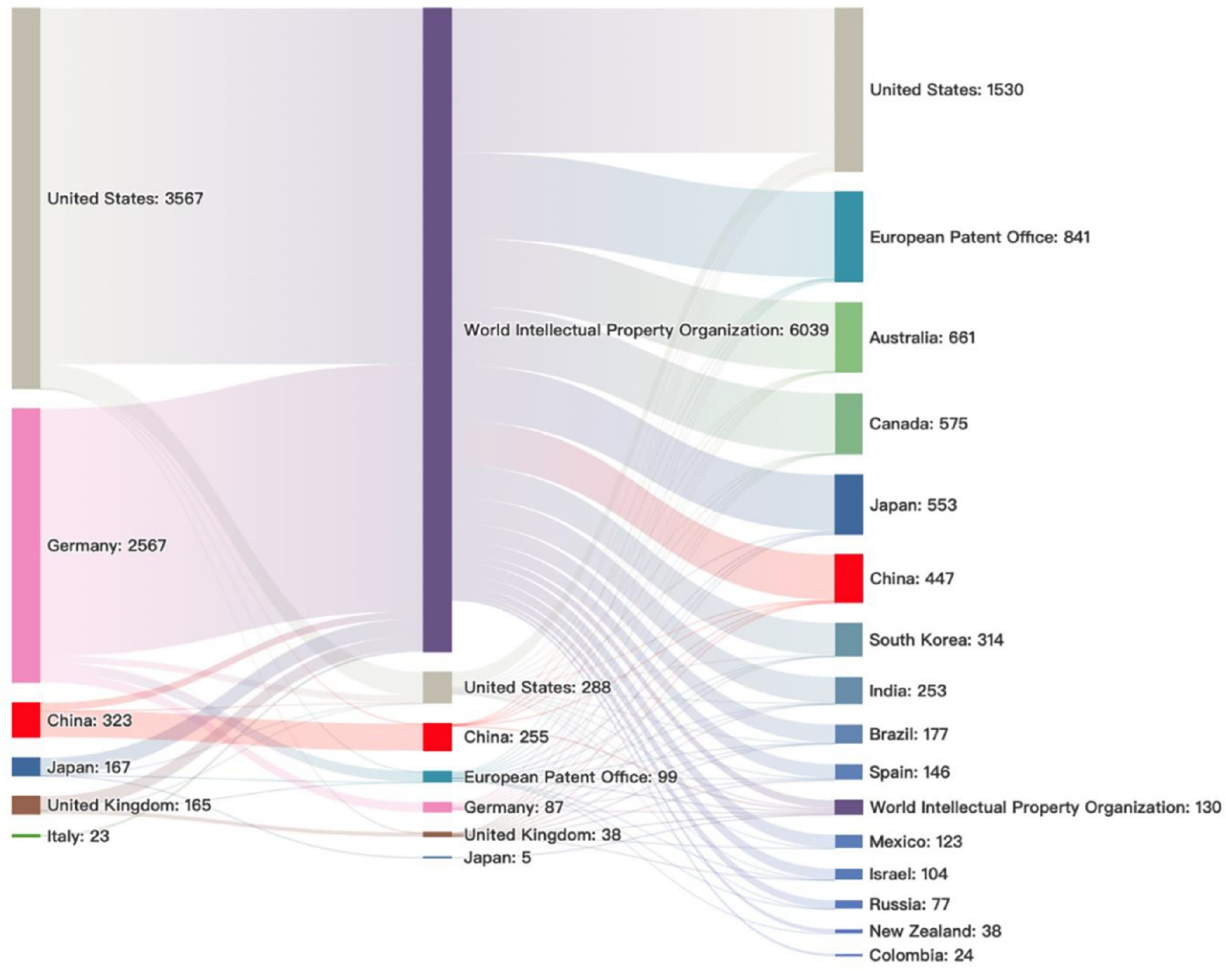
Flow of patent applications from the top six countries to patent offices around the world. Note: The column on the left is for home countries from which the patent assignees are located. The middle column represents the patent offices around the world that the priority patents in the patent families were filed to. The right column represents countries/regions where the intellectual properties are protected when those patents were applied through WIPO.

This report then examined the pattern of patent application flow related to technology development of mRNA therapeutics and vaccines. Our studies show that the U.S., Germany, the U.K., Japan, and Italy all have applied for technology protection in some overseas markets (Figure 7). In contrast, the majority of patents initiated by organizations in China were filed domestically to the Chinese Patent Office with some to the World Intellectual Property Organization, indicating that those Chinese organizations have placed less emphasis on seeking overseas protection of their intellectual properties. The U.S., Germany, the U.K., Japan, and Italy mainly distributed patents to other countries through the World Intellectual Property Organization. Among them, the number of patents distributed through the World Intellectual Property Organization in the U.S., Germany, Japan, and Italy accounted for more than 90%, and the number of patents distributed through the World Intellectual Property Organization in the U.K. accounted for more than 75.0%. The U.S. ranks first in the volume of patents distributed through the World Intellectual Property Organization, followed by Australia, Canada, Japan, and China. Countries such as the U.S., Australia, Canada, Japan, and China are also preferred when distributing patents through the European Patent Office.

## Major Organizations Contributing to the R&D of mRNA Therapeutics and Vaccines

### Organizations in Journal Publications

Among the top 15 organizations in the world publishing mRNA journal articles in this area (Table 1), 12 are from the U.S., indicating that the U.S. has a predominant role in basic research. Israel, the U.K., and China each has one organization ranking among the top 15. In terms of the nature of the top 15 organizations, 11 are universities, 3 scientific research institutions, and 1 company. The top three are Harvard University, the University of California, and the University of Pennsylvania, with 108, 84, and 84 journal articles, respectively. Tel Aviv University in Israel ranks fourth globally, with 74 papers. Johns Hopkins University, Moderna, Washington University, the U.S. Centers for Disease Control and Prevention, and the U.S. National Institutes of Health all ranked in the top 10. The University of Oxford in the U.K. ranks eighth in the world, with 57 articles. It may be worth mentioning that the U.S. Centers for Disease Control and Prevention and Mount Sinai Hospital account for more than 94% of the published journal articles in 2020–2022, indicating the high level of dedication of these two organizations to this area in recent years, probably related to the effort on fighting the COVID-19.

**Table 1:** Top 15 Organizations Publishing Journal Articles on RNA Therapeutics or Vaccines^a^

**Ranking**	**Organization**	**No. of journal publications**	**Country**	**Organization type**	**No. of journal publications in the past 3 years (% of total)**
1	Harvard University	108	U.S.	University	91 (84.3%)
2	University of California	84	U.S.	University	60 (71.4%)
2	University of Pennsylvania	84	U.S.	University	52 (61.9%)
4	Tel Aviv University	74	Israel	University	67 (90.5%)
5	Johns Hopkins University	71	U.S.	University	58 (81.7%)
6	Moderna	62	U.S.	Company	35 (56.5%)
7	Washington University	58	U.S.	University	39 (67.2%)
8	University of Oxford	57	U.K.	University	50 (87.7%)
9	Centers for Disease Control and Prevention	55	U.S.	Scientific research institution	52 (94.5%)
9	National Institutes of Health	55	U.S.	Scientific research institution	48 (87.3%)
9	Yale University	55	U.S.	University	50 (90.9%)
12	The University of Hong Kong	53	China	University	46 (86.8%)
13	Cornell University	51	U.S.	University	45 (88.2%)
13	Mount Sinai Hospital	51	U.S.	Scientific research institution	48 (94.1%)
13	Stanford University	51	U.S.	University	35 (68.6%)

a Data from the CAS Content Collection.

### Organizations in Patent Publications

Among the top 15 organizations with a high number of patents in this area (Table 2), the dominant organizations are mainly located in the U.S. (8 out of 15) followed by Germany (4 out of 15). Among these top patent applicants, companies are the main source of patents (11 out of 15). Thus, as expected, the major R&D effort of commercial companies has been focusing on the development of patentable technologies in this area. Moderna of the U.S. has produced most patents, followed by CureVac and BioNTech of Germany. The Chinese Academy of Science had the highest percentage of patents in the past three years, accounting for 70.6%, which may be indicative of an emphasis of R&D in this area by this organization in more recent years.

**Table 2:** Top 15 Organizations with Patent Applications on RNA Therapeutics or Vaccines^a^

**Ranking**	**Organizations**	**No. of patent applications**	**Country**	**Organization type**	**No. of patent applications in the past 3 years (% of total)**
1	Moderna	207	U.S.	Company	61 (29.5%)
2	CureVac	150	Germany	Company	15 (10.0%)
3	BioNTech	135	Germany	Company	59 (43.7%)
4	Translate Bio	78	U.S.	Company	48 (61.5%)
5	Tron	53	Germany	Company	11 (20.8%)
6	Alnylam Pharmaceuticals	30	U.S.	Company	2 (6.7%)
7	Shire Human Genetic Therapies	28	U.S.	Company	0 (0.0%)
8	University of Pennsylvania	27	U.S.	University	13 (48.1%)
9	Arcturus Therapeutics	23	U.S.	Company	8 (34.8%)
10	Acuitas Therapeutics	21	Canada	Company	5 (23.8%)
11	Chinese Academy of Sciences	17	China	Scientific research institution	12 (70.6%)
12	Massachusetts Institute of Technology	16	U.S.	University	4 (25.0%)
12	University of California	16	U.S.	University	6 (37.5%)
14	Ethris	15	Germany	Company	2 (13.3%)
15	Evox Therapeutics	14	U.K.	Company	4 (28.6%)

a Data from the CAS Content Collection.

## Patent Distribution among Key Technologies

**Figure 8:**
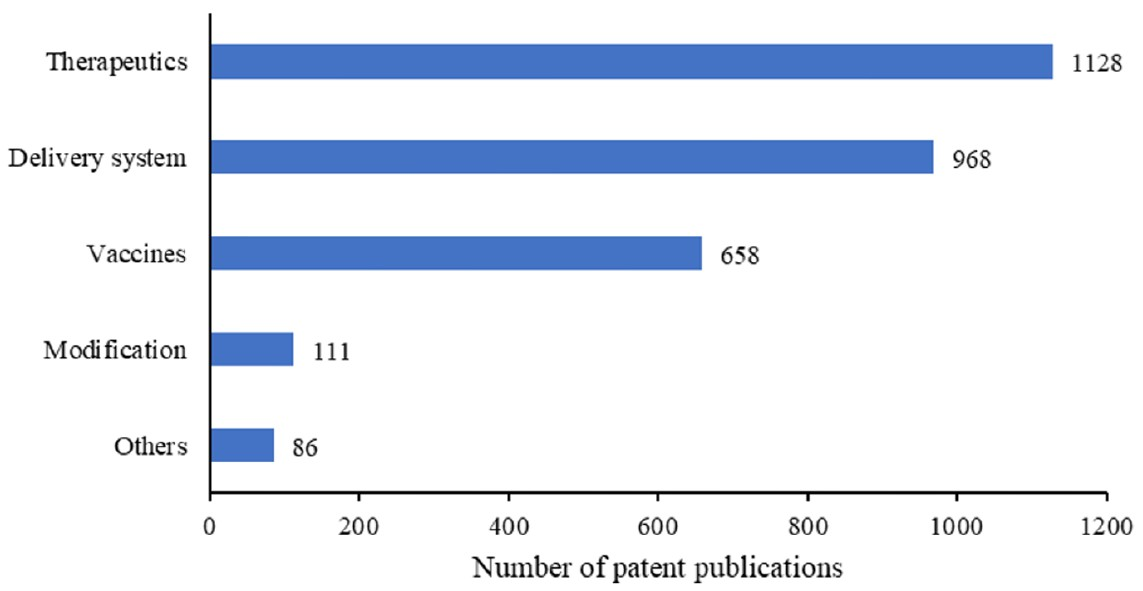
Distribution of patents among key classes of mRNA therapeutics and vaccines.

From the perspective of technology classification, mRNA patents mainly include therapeutic technology, delivery technology, vaccine technology, and mRNA modification technology (Figure 8). Out of 2,089 patents, 1,129 patents are related to the development of mRNA therapeutics for disease treatment, 977 related to development of delivery technology, and 659 related to vaccines. The total number is larger than 2,089 because some patents covered more than one specific technology area. The number of patent applications in these three types of technology account for 93.3% of the total patents, whereas the number of patents about mRNA modification technology is relatively small, though this technology is crucial to the success of mRNA vaccines and therapeutics.

### Analysis of Patents Related to mRNA Therapeutics

#### Top Countries/Regions with Published Patents Related to mRNA Therapeutics

**Figure 9:**
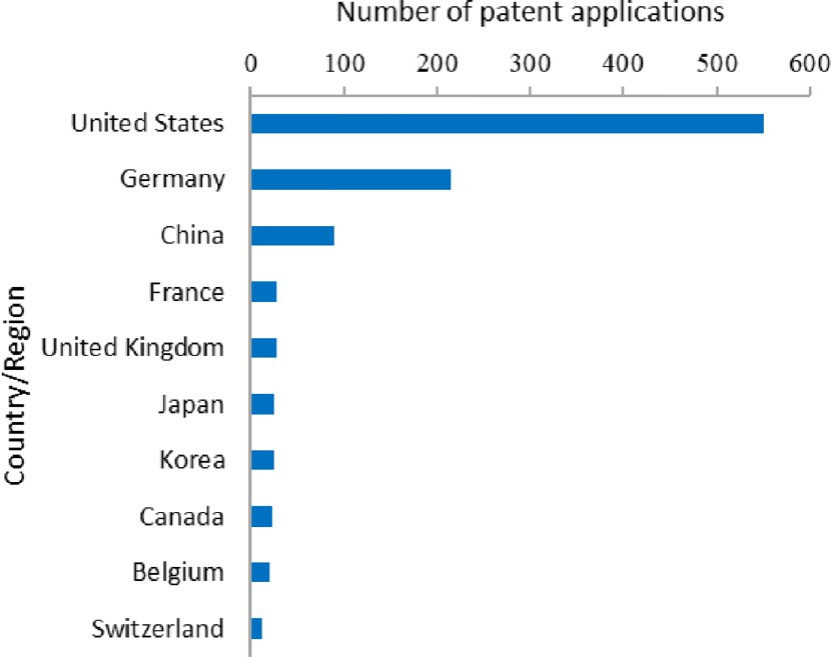
Distribution of the top 10 countries/regions in the global field of mRNA therapeutic patents.

Figure 9 shows the top 10 countries/regions where patent applicants in the field of mRNA therapeutics are located. The U.S. and Germany are the top two countries with 550 and 214 patents, respectively. The patents from these two countries account for about 67.7% of the total global patents in this area, reflecting the predominant role of these two countries in this area. China has filed 89 patents in this field, ranking distantly third.

#### Top Organizations with Published Patents Related to mRNA Therapeutics

Table 3 lists the top 11 organizations with patents related to mRNA therapeutics. As can be seen from the table, most of these organizations are in the U.S. (six) and Germany (four) with one in the U.K. Most of these patent applicants are commercial companies (nine) and only two are universities, indicating a heavy role of commercial companies in leading the R&D effort on mRNA therapeutics. The top five companies are Moderna, BioNTech, CureVac, Translate Bio, and Tron, with Moderna holding the largest number of patents (121).

**Table 3:** Top Patent Applicants for the Development of mRNA Therapeutics^a^

**Ranking**	**Organizations**	**No. of patent applications**	**Country**	**Organization type**
1	Moderna	121	U.S.	Company
2	BioNTech	92	Germany	Company
3	CureVac	79	Germany	Company
4	Translate Bio	36	U.S.	Company
5	Tron	34	Germany	Company
6	Shire Human Genetic Therapies	22	U.S.	Company
7	University of Pennsylvania	16	U.S.	University
8	The Ohio State University	15	U.S.	University
9	Arcturus Therapeutics	13	U.S.	Company
10	Ethris	10	Germany	Company
10	Evox Therapeutics	10	U.K.	Company
10	Massachusetts Institute of Technology	10	U.S.	University

a Data from the CAS Content Collection.

## Notable Patents Related to R&D of mRNA Therapeutics

Table 4 highlights several of the most notable patents focused on the development of mRNA therapeutics.

**Table 4:** Notable Patents on mRNA Therapeutics

**Patent number**	**Organization**	**Patent title**
WO2013096709	Moderna, USA	Increasing the viability or longevity of an organ or organ explant using modified mRNAs for proteins essential for organ survival
WO2015058069	Moderna, USA	Polynucleotides for tolerizing cellular systems
WO2016201377	Moderna, USA	Preparation of targeted adaptive vaccines for treatment of inflammatory disease, autoimmune disease and cancers
WO2017214175	Moderna, USA	Modified RNA encoding VEGF-A in formulations for treatment of heart failure and other diseases
WO2018160540	Sanofi, France; BioNTech, Germany	Therapeutic RNA and uses in treating solid tumor cancers
WO2018222890	Arcturus Therapeutics, USA	Synthesis and structure of high potency RNA therapeutics
WO2019178006	SQZ Biotechnologies Co., USA	Immunogenic epitope and adjuvant-modified T cells for intracellular delivery of tumor or exogenous antigen to enhance immune response against cancer and infection
WO2020056147	Moderna, USA	Polynucleotides encoding glucose-6-phosphatase for the treatment of glycogen storage disease
WO2020097409	Moderna, USA	Use of mRNA encoding OX40L in combination with immune checkpoint inhibitor to treat cancer in human patients
WO202011811	Arcturus Therapeutics, USA	Compositions and methods for treating ornithine transcarbamylase deficiency
WO2020154189	Sanofi, France	Therapeutic RNA for treatment of advanced stage solid tumor
WO2020227615	Moderna, USA	Polynucleotides encoding methylmalonyl-CoA mutase for the treatment of methylmalonic acidemia
WO2020260685	eTheRNA Immunotherapies, Belgium	Antitumor therapy comprising mRNA molecules encoding tumor-associated antigens and checkpoint inhibitors
WO2021021988	Translate Bio, USA	Treatment of cystic fibrosis by delivery of nebulized mRNA encoding Cystic Fibrosis Transmembrane Conductance Regulator (CFTR)
WO2021058472	BioNTech and TRON, Germany	Combination treatment using therapeutic antibody and interleukin 2 (IL-2)
WO2021198157	BioNTech, Germany	mRNA compositions (RiboMab) expressing claudin-18.2-targeting antibody and anticancer uses thereof
WO202120771	Verve Therapeutics, USA	Base editing of ANGPTL3 and methods of using same for treatment of cardiovascular disease
WO2021214204	BioNTech, Germany	RNA constructs and uses thereof
WO2022136266	BioNTech, Germany	Therapeutic RNA for treating cancer

Patent application WO2020097409 by Moderna, USA features methods for treating ovarian cancer, as well as other cancers such as solid tumors, lymphomas, and epithelial origin cancers, by administering mRNA encoding an OX40L polypeptide, also known as CD252. The disclosure also presents pharmaceutical composition for intratumoral administration comprising lipid nanoparticles with a mRNA encoding a human OX40L. The disclosure also features combination therapies, such as the use of mRNA encoding an OX40L polypeptide in combination with a checkpoint inhibitor, such as an anti-PD-L1 antibody.

Patent application WO2021198157 by BioNTech, Germany, provides RNA technologies for targeting Claudin-18.2 (CLDN-18.2) polypeptides. Such RNA technologies can be useful for the treatment of Claudin-18.2 pos. cancer, including biliary cancers, ovarian cancers, gastric cancers, gastroesophageal cancers, and pancreatic cancers. Noteworthy is a mRNA formulation encoding monoclonal IgG1, such as Zolbetuximab (Claudiximab).

Patent application WO2018160540 by Sanofi, France, and BioNTech, Germany, relates to the field of therapeutic mRNAs for the treatment of solid tumors, including medical preparation comprising mRNA encoding an IL-12sc protein, an IL-15 sushi protein, an IFNα protein, and a GM-CSF protein. The disclosed pharmaceutical formulations are for use in a method of preventing cancer metastasis.

Patent application WO2020260685 by eTheRNA Immunotherapies, Belgium, relates to combinations of mRNAs encoding CD40, caTLR4, and CD70 with mRNAs encoding tumor-associated antigens for use as therapeutic vaccine in the treatment of metastatic cancer patients, primarily with malignant melanoma disease, but also to other cancer types. The disclosed therapies may further encompass the administration of checkpoint inhibitors. The invention provides administration schemes focusing on administration of the therapeutic into lymph nodes, the so-called intranodal therapy.

### Analysis of Patents Related to mRNA Vaccines

#### Top Countries/Regions with Published Patents Related to mRNA Vaccines

**Figure 10:**
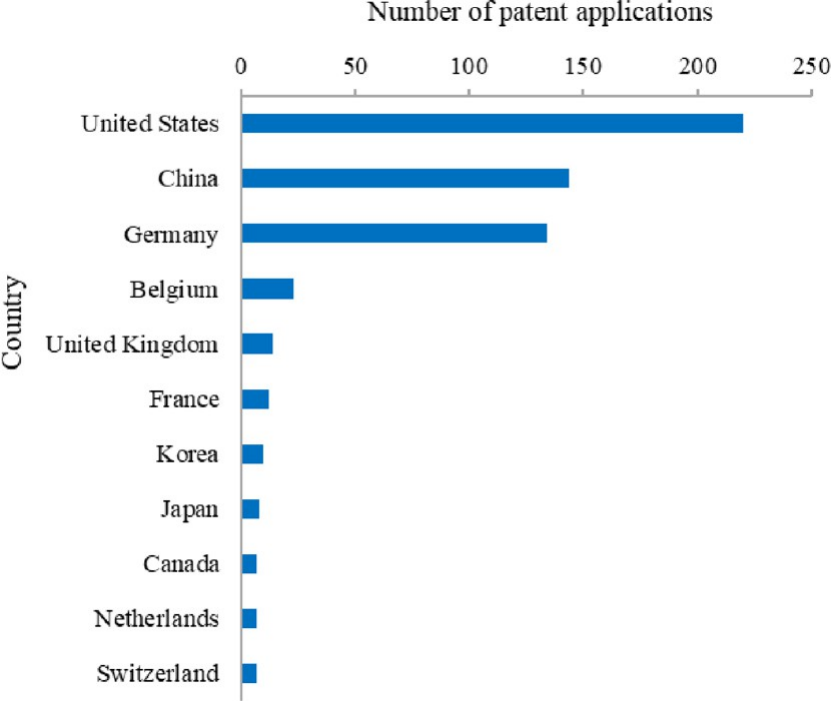
Distribution of mRNA vaccine patents among countries/regions with the largest numbers of patents.

The patent applicants in the global mRNA vaccine field are mainly from the U.S., China, Germany, and countries and regions shown in Figure 10. The U.S., China, and Germany have 220, 144, and 134 patent applications respectively, making them the three countries with the strongest strength in this technology field. It needs to point out that China does not yet have a mRNA vaccine on market probably due to its late involvement in this area and thus the patent data here does not necessarily reconcile with the market data and correlate with the impact. Countries such as Belgium, the U.K., and France have less than 30 patent applications in this field.

### Distribution of mRNA Vaccine Patents among Top Organizations

Table 5 lists the top 12 organizations in terms of mRNA vaccine patent output. Among them, five are from the U.S., three from Germany, two from China, and one each from the U.K. and Belgium. Like other technical fields, companies are the main contributors of mRNA vaccine patents (8 out of 12). The top 3 companies are CureVac (92), Moderna (59), and BioNTech (38).

**Table 5:** Global Patent Applications by Major Institutions in the Field of mRNA Vaccines^a^

**Order**	**Organization**	**No. of patent applications**	**Country**	**Organization type**
1	CureVac AG	92	Germany	Company
2	Moderna	59	U.S.	Company
3	BioNTech	38	Germany	Company
4	TRON GmbH	21	Germany	Company
5	Chinese Academy of Sciences	10	China	Scientific research institution
6	GlaxoSmithKline Biologicals S.A.	10	U.K.	Company
7	Translate Bio	7	U.S.	Company
8	CanSino Biologics	6	China	Company
8	eTheRNA Immunotherapies	6	Belgium	Company
8	University of California	6	U.S.	University
8	University of Florida	6	U.S.	University
8	University of Pennsylvania	6	U.S.	University

a Data from the CAS Content Collection.

### Most Notable Patents Related to mRNA Vaccine R&D

Over 650 patents related to mRNA vaccine development with their associated information were identified in this study. Tables 6 and 7 list several intriguing patents focused on the mRNA vaccines for infectious diseases and cancers, respectively, the two major disease classes for which mRNA vaccines are being developed.

**Table 6:** Notable Patents Focused on the Development of mRNA Vaccines for Infectious Diseases

**Patent number**	**Organization**	**Patent title**
WO2021213924	BioNTech, Germany	Coronavirus RNA vaccine encoding SARS-CoV-2 spike protein for preventing COVID-19
WO2020190750	Moderna, U.S. Dept. of Health and Human Services, USA	Preparation of HIV Env- and lentivirus Gag protein-encoding mRNA VLP vaccine to induce broad-spectrum neutralizing antibodies for treating HIV infection
WO2018151816	Moderna, USA	Immunogenic compositions for Zika virus including cationic lipid nanoparticles encapsulating mRNA having an open reading frame encoding a viral, bacterial or parasitic antigen, a pan HLA DR-binding epitope (PADRE) and a 5′ terminal cap modified to increase mRNA translation efficiency
WO2021155243	Moderna, USA	Respiratory virus vaccine compositions
WO2013055905	Novartis, Switzerland	Recombinant self-replicating polycistronic RNA molecules expressing multiple herpes virus proteins and their use in vaccines for inducing neutralizing antibodies
WO2021159040	Moderna, USA	Engineering SARS CoV-2 mRNA vaccines expressing key neutralizing domains of spike protein, individually or in combination, for inducing protective immunity and immunotherapy
WO2021251453	Daiichi Sankyo, University of Tokyo, Japan	Nucleic acid lipid particle vaccine encapsulated with severe acute respiratory syndrome coronavirus 2 messenger ribonucleic acid
WO2021159130	Moderna, USA; U.S. Dept. of Health and Human Services, USA	Preparation of SARS CoV-2 mRNA vaccines encoding full-length spike protein variant, stabilized into a prefusion conformation, encapsulated in a lipid nanoparticle formulation
WO2021255270	Ziphius Vaccines, Belgium; Universiteit Gent, Belgium	Self-amplifying COVID-19 RNA vaccine encoding SARS-CoV-2 Spike and Nucleocapsid protein antigen and alphavirus Nonstructural protein
WO2017070613	Moderna, USA	Human cytomegalovirus RNA vaccines
WO2021204179	Suzhou Abogen Biosciences, China	Nucleic acid vaccines for coronavirus
WO2021226436	Translate Bio, USA; Sanofi, France	Optimized nucleotide sequences encoding SARS-COV-2 antigens
WO2017070623	Moderna, USA	Herpes simplex virus RNA vaccine
WO2021160346	Institut Pasteur, France	Nucleic acid vaccine against severe acute respiratory syndrome coronavirus SARS-CoV-2
WO2022171182	Stemirna Therapeutics, China	Vaccine reagent for treating or preventing coronavirus mutant strain
CA3132188	Providence Therapeutics Holdings, Canada	Compositions and methods for the prevention and/or treatment of COVID-19
WO2021183563	Arcturus Therapeutics, USA	Coronavirus vaccine compositions and methods
WO2022150717	Moderna, USA	Seasonal RNA influenza virus vaccines
WO2022129918	Imperial College, UK Innovations Ltd, U.K.	Engineering a thermally stabilized self-amplifying RNA vaccine based on Venezuelan Equine Encephalitis virus backbone encoding SARS-CoV-2 spike glycoprotein encapsulated in lipid nanoparticle formulation for preventing and/or treatment of COVID-19
WO2022178196	Sanofi Pasteur, USA	Meningococcal B recombinant vaccine
WO2022137133	CureVac, Germany; GlaxoSmithKline Biologicals SA, U.K.	RNA vaccine against SARS-CoV-2 variants
WO2022116528	Suzhou CureMed Biomedical Technology, China	Circular RNA vaccine containing circular RNA and kit for detecting novel coronavirus neutralizing antibody
US20220325255	University of Texas, USA	Compositions and methods for treating viral infections targeting TRIM7

**Table 7:** Notable Patents Focused on the Development of mRNA Vaccines for Cancer

**Patent number**	**Organization**	**Patent title**
WO2021155149	Genentech, USA; BioNTech SE, Germany; F. Hoffmann-La Roche, Switzerland	Methods of inducing neoepitope-specific T cells with a PD-1 axis binding antagonist and an RNA vaccine
WO2015024664	CureVac, Germany	Composition comprising mRNA encoding a combination of tumor antigens as vaccine for treating prostate cancer
WO2012019168	ModernaTX, USA	Use of modified mRNA encoding melanocyte stimulating hormone, insulin and granulocyte colony-stimulating factor in prevention or treatment of disorders
WO2020097291	ModernaTX, USA	Cancer vaccines comprising mRNA(s) encoding peptide epitopes (neoepitopes) and formulated as lipid nanoparticles
WO2020141212	eTheRNA Immunotherapies NV, Belgium	mRNA vaccine
WO2022008519	BioNTech SE, Germany; TRON – Translationale Onkologie Mainz, Germany	Therapeutic RNA for HPV-positive cancer
WO2015024666	CureVac, Germany	RNA vaccine for treating lung cancer
WO2012159643	BioNTech AG, Germany; TRON – Translationale Onkologie Mainz, Germany	Individualized vaccines for cancer
WO2015014869	BioNTech AG, Germany; TRON – Translationale Onkologie Mainz, Germany	Determination of expression pattern of a set of tumor antigens including Cxorf61, CAGE1, PRAME and others to select cancer therapy regimen
WO2022009052	Janssen Biotech, USA	Prostate neoantigens and their uses
WO2022081764	RNAimmune, USA	Pan-ras mRNA cancer vaccines
WO2014082729	BioNTech AG, Germany; Mainz Gemeinnuetzige GmbH, Germany	Individualized vaccines for cancer
WO2016180467	BioNtech Cell & Gene Therapies, Germany; TRON – Translationale Onkologie Mainz, Germany	Enhancing the effect of car-engineered T cells by means of nucleic acid vaccination

Patent application WO2021255270 (Ziphius Vaccines, Belg.; Universiteit Gent) discloses a self-amplifying COVID-19 vaccine comprising a mRNA encoding SARS-CoV-2 spike protein, nucleocapsid protein, and alphavirus nonstructural proteins (nsp1–4) in thermally stabilizing RNA vaccine formulation. Patent application WO2022129918 (Imperial College Innovations Limited, U.K.) discloses novel uses and methods for thermally stabilizing RNA vaccine formulations, including self-amplifying RNA replicons derived from Venezuelan Equine Encephalitis virus encoding SARS-CoV-2 spike protein encapsulated in lipid nanoparticles. The higher and prolonged in vivo translation improves the efficacy of self-amplifying RNA vaccines even in low dose.

Patent application WO2022137133 (CureVac AG and GlaxoSmithKline Biologicals) discloses that the mRNA vaccine encoding variants of highly immunogenic SARS-CoV-2 spike protein in lipid nanoparticle formulation induces neutralizing antibodies and immune cell responses against SARS-CoV-2.

Patent application WO2021155243 (Moderna) discloses a vaccine comprising a codon optimized human respiratory syncytial virus (hRSV) nucleic acid encoding a stabilized prefusion form of an hRSV F glycoprotein variant formulated in the lipid nanoparticles. In vivo study was conducted to evaluate the immunogenicity, efficacy, and safety of the mRNA vaccine in mice and the RSV cotton rat model.

Patent application WO2022116528 (Suzhou CureMed Biomedical Technology) discloses a circular RNA (circRNA) vaccine comprising a specific internal ribosome entry site (IRES) element and receptor domain of SARS-CoV-2 spike protein without 5′ or 3′ ends. The covalently closed structure of circRNA prevents the degradation by exonucleases and improves its biostability. They exemplified the further application for making circRNAs encoding erythropoietin, anti-PD1 antibody, interleukin 15, prostate cancer specific antigen PAP, and CD16 CAR receptor for protein expression in 293T cells.

Patent application US20220325255 (University of Texas) discloses antiviral compositions including mRNA encoding for a TRIM7 protein encapsulated into a lipid nanoparticle (LNP), as well as methods for impairing enterovirus replication for treating viral infections. The disclosure provides for the first time E3 ligase targeting an enterovirus protein and the first demonstration that a viral membrane remodeling protein is subject to degradation as a host antiviral strategy.

Patent application WO2021155149 by Genentech, BioNTech, and Hoffmann-La Roche AG discloses mRNA vaccines composed of mRNAs encoding up to 20 neoepitopes (two decatopes) deriving from cancer-specific mutations in patients formulated in cationic liposomes (Table 7). The first-in-human phase Ia and Ib studies of a mRNA vaccine as a monotherapy and in combination with atezolizumab were conducted in patients with advanced or metastatic solid tumors. They showed innate and neoantigen-specific immune responses induced by the mRNA vaccine alone and combined with atezolizumab.

Patent application WO2015024664 by CureVac discloses development of a mRNA-based personalized cancer vaccine encoding prostate cancer-associated antigens, prostate-specific antigen), PSMA (prostate-specific membrane antigen), PSCA (prostate stem cell antigen), STEAP (six transmembrane epithelial antigen of the prostate), MUC1 (mucin 1) and PAP (prostatic acid phosphatase) for treating prostate cancer.

Patent application WO2016180467 by BioNtech and TRON Translationale Onkologie Mainz discloses administering to the mammal T cells genetically modified to express a chimeric antigen receptor (CAR) targeted to antigen. Antigen is selected from claudin 18.2, claudin 6, CD19, CD20, CD22, CD33, CD123, mesothelin, CEA, c-Met, PSMA, GD-2, or NY-ESO-1. CAR-transgenic human CD8+ T cells targeting claudin-6 proliferated in response to CLDN6-transfected autologous dendritic cells. Murine CLDN6-CAR T cells were able to proliferate strongly in response to murine BMDCs expressing human CLDN6 antigen after RNA transfer.

Patent application WO2020097291 by Moderna discloses mRNA cancer vaccines composed of mRNAs encoding 3–50 neoepitopes formulated in cationic lipid nanoparticles. A phase I study was undertaken to assess the safety, tolerability, and immunogenicity of mRNA vaccine monotherapy in patients with resected solid tumors and in combination with pembrolizumab in patients with unresectable solid tumors. A randomized phase II clinical study was conducted in patients with resected cutaneous melanoma.

## Analysis of Patents Related to mRNA Delivery Systems

### Top Countries/Regions with Published Patents Related to mRNA Delivery Systems

**Figure 11:**
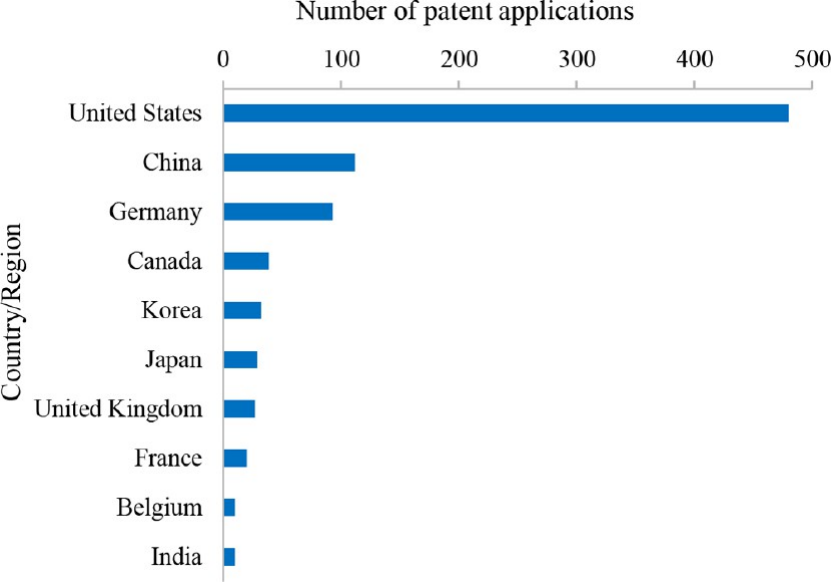
Distribution of patents among the top 10 countries/regions in the field of mRNA delivery systems.

Patent applications in the field of mRNA delivery systems are mainly from the U.S., China, Germany, and other countries, as shown in Figure 11. The U.S. has the largest number of patents (480), which equals to almost the combined number of patents from other countries.

### Top Organizations with Published Patents Related to mRNA Delivery Systems

As shown in Table 8, like that in the field of mRNA therapeutics, global patent applicants in the field of mRNA delivery systems are primarily located in the U.S. (6 of top 10), Germany (2 out of 10)), Canada (1 out of 10), and the U.K. (1 out of 10. In terms of the nature of organizations, companies are the main source of these patents (9 out of 10), The top 5 patent applicants are Modena, Translate Bio, CureVac, Alnylam Pharmaceuticals, and BioNTech with Modena having the largest number of patents (64).

**Table 8:** Top Patent Applicants in the Field of mRNA Delivery Systems^a^

**Number**	**Organizations**	**No. of patent applications**	**Country**	**Organization type**
1	Moderna	64	U.S.	Company
2	Translate Bio	45	U.S.	Company
3	CureVac AG	30	Germany	Company
4	Alnylam Pharmaceuticals	24	U.S.	Company
5	BioNTech	22	Germany	Company
6	Acuitas Therapeutics	19	Canada	Company
7	Shire Human Genetic Therapies	15	U.S.	Company
8	Massachusetts Institute of Technology	13	U.S.	University
9	Arcturus Therapeutics	12	U.S.	Company
9	Evox Therapeutics Ltd.	12	U.K.	Company

a Data from the CAS Content Collection.

### Key Technology Layout in the Field of mRNA Modification

From the annual trend, the number of global patent applications in the field of mRNA modification fluctuates, with a significant increase and peak in 2013, followed by a decline (Supplemental Figure S3). The trend of patent applications was relatively stable from 2015 to 2018, and after a brief decline in 2019, the number of patent applications showed a slow growth trend from 2020.

From the distribution of patent research topics, the global field of mRNA modification focuses on drug delivery types, carrier materials, nucleic acid modification, mechanism research, and other aspects (Supplemental Figure S4). Among them, in terms of the type of administration, key concepts include intraperitoneal injections, pharmaceutical intravenous injections, subcutaneous injections, etc. With regard to carrier materials, nanoparticles are infused with cationic lipids, pharmaceutical nanoparticles, pharmaceutical liposomes, etc. Nucleic acid modifications include oligonucleotide analogs, nucleotide analogs, peptide nucleic acids, etc. In terms of mechanism research, key concepts include transcription, growth factors, membrane proteins, etc.

From the perspective of patent origin countries, the patentees in the global field of mRNA modification are mainly from the U.S., Germany, China, Japan, and France (Supplemental Figure S5). Among them, the U.S. filed the largest number of patents in the field, Germany and China are in second and third places, with Japan and France last filing a relatively small numbers of patents.

From the perspective of patent application institutions, global patent application institutions in the field of mRNA modification are mainly concentrated in the U.S. and Germany (Supplemental Table S1). Among the top 10 patent-filing organizations, six are from the U.S. and four are from Germany. From the perspective of institutional nature, companies are still the main body of patent application, accounting for about 70% of all applications. The top five patent filers were Modena, CureVac, BioNTech, Alnylam Pharmaceuticals, and Translate Bio, which also ranked among the top 5 in the field of mRNA modification.

## Notable Patents on mRNA Delivery and Modification

RNAs, which are hydrophilic and negatively charged, cannot diffuse across cell membranes; thus, they require delivery vectors and/or chemical modification to reach their targets. mRNAs may also be quickly hydrolyzed by circulating RNases. As such, when administered systemically, RNA delivery systems need to protect the RNA against serum nucleases, bypass the undesirable immune reaction against mRNA per se, avoid nonspecific interactions with serum proteins, and block renal clearance.^47^ Thus, delivery vehicles and chemical modifications are of the utmost importance for the success of the mRNA therapeutics. Table 9 summarizes some notable patents disclosing essential advances in these areas.

**Table 9:** Notable Patents Focused on mRNA Delivery and Modification

**Patent number**	**Organization**	**Patent title**	**Disclosure highlight**
WO2013086373	Alnylam Pharmaceuticals, USA	Lipids for the delivery of nucleic acids	LNP components for RNA delivery
WO2014093924	Moderna, USA	Preparation, cytotoxicity, apoptosis, and transcription of modified nucleic acid molecules and uses thereof	Modification of mRNA
WO2016070166	Arcturus Therapeutics, USA	Translatable messenger RNA analogs containing unlocked nucleomonomers and with prolonged in vivo half-lives for therapeutic uses	mUNA or mRNA analogs with unlocked nucleomonomers
US10808242	BioNTech, Germany	Method for reducing immunogenicity of RNA by constructing A-rich and U-poor mRNA for use in therapy	mRNA modifications for decreasing nonspecific immunogenicity by mRNA itself
WO2017117528	Acuitas Therapeutics, Canada	Preparation of lipids and lipid nanoparticle formulations for delivery of nucleic acids	LNP components and formulation for nucleic acid delivery
WO2017176974	The Ohio State University, USA	Biodegradable amino-ester nanomaterials for nucleic acid delivery	LNPs for delivery of RNAs including siRNA, miRNA, and mRNA
WO2017212009	Curevac AG, Germany	Hybrid carriers comprising cationic peptide or polymer and lipidoid	A nucleic acid delivery system comprised of a cationic peptide or polymer and a lipidoid compound
US20180125989	Translate Bio, USA	Imidazole cholesterol ester (ICE)-based lipid nanoparticle formulation for delivery of mRNA	Methods of formulating nucleic acid containing LNP
WO2018009838	Rubius Therapeutics, USA	Compositions and methods related to therapeutic erythroid cell systems expressing exogenous RNA encoding a protein	Therapeutic erythroid cell systems expressing exogenous RNA encoding a protein
WO2018013525	Translate Bio, USA	Nucleic acid conjugates and uses thereof	Conjugates comprising sugars, folates and cell-penetrating peptides for delivering mRNA
WO2019092145	Evox Therapeutics, U.K.	Exosomes comprising RNA therapeutics	Methods for using extracellular vesicles to encapsulating nucleic acid-based therapeutics such as mRNA, circular RNA, miRNA, etc.
WO2020070040	Johannes Gutenberg-University Mainz and BioNTech, Germany	RNA particles comprising polysarcosine	LNPs for delivering mRNAs
WO2020061367	Moderna, USA	Preparation of compounds and lipid nanoparticle compositions for intracellular delivery of therapeutic agents	LNPs for drug delivery
WO2020097540	Arbutus Biopharma Corp., Canada	Methods and lipid nanoparticles for delivering mRNA and siRNA in treatment of diseases	LNPs for mRNA delivery
WO2020263883	Moderna, USA	Endonuclease-resistant messenger RNA and uses thereof	Chemically modified mRNA that increases mRNA stability
CN110747214	Shenzhen Zhenzhi Medical Technology, China	Preparation of mRNA-antibody fusion molecule and its use for drug delivery	Preparation of antibody–mRNA fusion/conjugate with puromycin as the linker for targeted delivery of mRNA therapeutics.
WO2020160397	Moderna, USA	Methods of preparing lipid nanoparticles	LNP formulation
WO2021001417	BioNTech, Germany	RNA formulations suitable for therapy	Self-amplifying RNA formulated in various polymers
WO2021231854	Moderna, USA	Lipid nanoparticle compositions comprising an mRNA therapeutic and an effector molecule	System that features a tethered molecule to further increase the level and/or activity of mRNA therapeutics formulated in LNP
WO2021257262	Yale University, USA	Poly(amine-co-ester) polymers with modified end groups and enhanced pulmonary delivery	PEGlyated poly(amine-co-ester) polymers with modified end groups for enhanced delivery of mRNA to the lung by inhalation
WO2022032154	Moderna, USA	Compositions for the delivery of payload molecules to airway	LNPs comprising payload molecules such as mRNA therapeutics to be delivered to airway cells
WO2016176330	University of Pennsylvania, USA; Acuitas Therapeutics, Canada	Nucleoside-modified mRNAs encoding antigens for inducing an adaptive	Modified antigen mRNA delivered in LNP induced adaptive immune response without inducing innate immunity
WO2020191103	Arcturus Therapeutics, USA	Method of making lipid-encapsulated RNA nanoparticles	Detailed method for making RNA-encapsulating LNP
WO2021250263	eTheRNA Immunotherapie, Belgium	Lipid nanoparticles comprising ionizable lipid, phospholipid, sterol, PEG lipid and mRNA	LNP components for RNA delivery
WO2022175815	Pfizer, USA	Methods of protecting RNA	Methods of protecting RNA against degradation and components comprising free amino acids for this purpose
WO2019246203	University of Texas, USA	Lipid nanoparticle compositions for delivery of mRNA and long nucleic acids	Compositions for delivery of long nucleic acids (>80 nucleotides), such as mRNAs, including cationic ionizable lipid, phospholipid, PEGylated lipid, and a steroid
WO2022236093	Carnegie Mellon University, USA	Lipid nanoparticle-mediated mRNA delivery to the pancreas	LNP composition for mRNA delivery to the pancreas containing: cationic helper lipid, cholesterol analog, PEG-based compound, ionizable lipidoid, and mRNA
US20230043677	Oregon State University, USA	Inhalable therapeutics	Nanoparticles for mRNA delivery suitable for nebulization and/or delivering mRNA by inhalation
WO2022155598	Tufts College and Brigham and Women’s Hospital, USA	Lipid nanoparticles for targeted delivery of mRNA	LNP composition for specific delivery of CRISPR-Cas9 mRNA to the lung or liver

Patent application WO2013086373 by Alnylam Pharmaceuticals, USA, relates to novel cationic lipids that can be used in combination with other lipid components such as a neutral lipid, a sterol such as cholesterol, and a PEG-lipid conjugate capable of reducing aggregation, to form lipid nanoparticles with oligonucleotides, to facilitate the cellular uptake and endosomal escape, and to knockdown target mRNA both in vitro and in vivo. Exemplary LNP composition comprised 50% cationic lipid, 10% DSPC, 38.5% cholesterol, and 1.5% PEG-DMG (average PEG molecular weight of 2000).

Patent application WO2020160397 by Moderna, USA, provides methods of producing LNP formulations and the produced LNP formulations thereof. It reflects the recent efforts toward “bedside” and/or “point-of-care” formulations, whereby mRNA may be encapsulated within preformed vesicles that were prepared at an earlier date. This mode of production offers advantages in the context of clinical supply, as empty LNP vesicles may be produced and stored separately prior to recombination with mRNA in a clinical compound setting. Specifically, bedside formulations may promote increased stability, since mRNA and empty raw materials can be stored in separately optimized conditions. Process complexity and cost of goods may be reduced since the LNP preparation occurs independent of cargo, enabling a platform approach for multiple mRNA or active agent constructs. The empty LNP plus mRNA modality may be referred to as “post-hoc”. The concept of post hoc loading as described in the present invention may enable control and/or optimization of each step separately. Further, the post hoc loading may enable mRNA addition at timescales that enable point-of-care formulation, e.g., months or years following empty LNP production.

Patent application WO2017176974 by The Ohio State University, USA, relates to biodegradable amino-ester lipid nanoparticles for efficient delivery of RNAs including siRNA, miRNA, and mRNA. Provided are also compositions including an amino-ester lipid compound of the invention, a noncationic lipid, a PEG-lipid conjugate, a sterol, and an active agent such as mRNA, which can be used to correct a mutation in a genome. For example, mRNAs can be delivered to correct mutations that cause hemophilia due to mutations in the genes encoding Factor VIII (hemophilia A) or Factor IX (hemophilia B).

Patent application WO2019092145 by Evox Therapeutics, UK, pertains to extracellular vesicles (Evs), specifically exosomes, as delivery vehicles for nucleic acid-based therapeutics. The distinctive properties of the extracellular vesicles (Evs), and specifically their nanosized subgroup, the exosomes—their innate stability, low immunogenicity, biocompatibility, and good biomembrane penetration capacity—allow them to function as superior natural nanocarriers for efficient drug delivery and are currently viewed as the rising star in drug delivery.^48^ The nucleic acid therapeutics of the present invention are loaded into Evs using inventive engineering protein and nucleic acid engineering strategies to enhance loading into Evs and to facilitate release of the nucleic acid cargo molecules inside target cells.

Patent application US10808242 by BioNTech, Germany, is focused on decreasing immunogenicity of RNA. The provided methods for decreasing immunogenicity of RNA comprise modifying the nucleotide sequence of the RNA by reducing the uridine (U) content, by elimination of U nucleosides from the nucleotide sequence of the RNA and/or a substitution of U nucleosides by nucleosides other than U in the nucleotide sequence of the RNA. Using RNA having decreased immunogenicity allows administration of RNA as a drug to a subject, e.g., in order to obtain expression of a pharmaceutically active peptide or protein, without eliciting an immune response which would interfere with therapeutic effectiveness of the RNA or induce adverse effects in the subject.

Patent application WO2020069718 by Johannes Gutenberg-University Mainz and BioNTech, Germany, relates to RNA particles for delivery of RNA to target tissues after parenteral administration and compositions comprising such RNA particles. Specifically, polysarcosine–lipid conjugates are featured as suitable components for the assembly of RNA nanoparticles. By now, PEG has been the most widely used and gold standard “stealth” polymer in drug delivery. However, PEG has been found to exhibit some undesired effects such as lowering transfection efficiency, accelerated blood clearance induced by anti-PEG antibodies, and/or complement activation, as well as inducing a specific immune response. The present invention shows that polysarcosine-lipid conjugates avoid the disadvantages accompanied by the use of PEG. Polysarcosine–lipid conjugates enable manufacturing of RNA nanoparticles with different techniques, resulting in defined surface properties and controlled size ranges. Manufacturing can be done by robust processes that are compliant with the requirements for pharmaceutical manufacturing. The particles can be end-group functionalized with different moieties to modulate charge or to introduce specific molecular moieties like ligands.

Patent application US20230043677 by the Oregon State University, USA, relates to nanoparticle composition for encapsulating a therapeutic agent, such as a mRNA, suitable for nebulization and/or delivery of the nebulized formulation to the lungs by inhalation. The nanoparticles comprise an ionizable lipid, a cholesterol derivative, a structural lipid, and a PEG lipid.

Patent application WO2022155598 by Tufts College and Brigham and Women’s Hospital, USA, discloses a highly potent nonviral LNP-mediated CRISPR-Cas9 delivery system for liver or lung delivery of Cas9 mRNA, and demonstrates its efficacy by targeting the *Angptl3* gene. The system is composed of a leading tail-branched bioreducible lipidoid (306-012B) co-formulated with an optimized mixture of excipient lipid molecules, and it successfully co-delivers SpCas9 mRNA and a single guide RNA targeting *Angptl3* via a single administration.

The success of mRNA-based COVID-19 vaccines have demonstrated the effectiveness of two key strategies for developing RNA medications: the chemical modifications of mRNA uridine to pseudouridine and 5′-capping, as well as the lipid nanoparticle delivery vectors, paving the way for further advancement of mRNA therapeutics and vaccines.

## Application of mRNA Therapeutics and Vaccines in Disease Treatment and Prevention

### Analysis of Diseases Claimed in Patents Related to mRNA Therapeutics and Vaccines among Diseases

**Figure 12:**
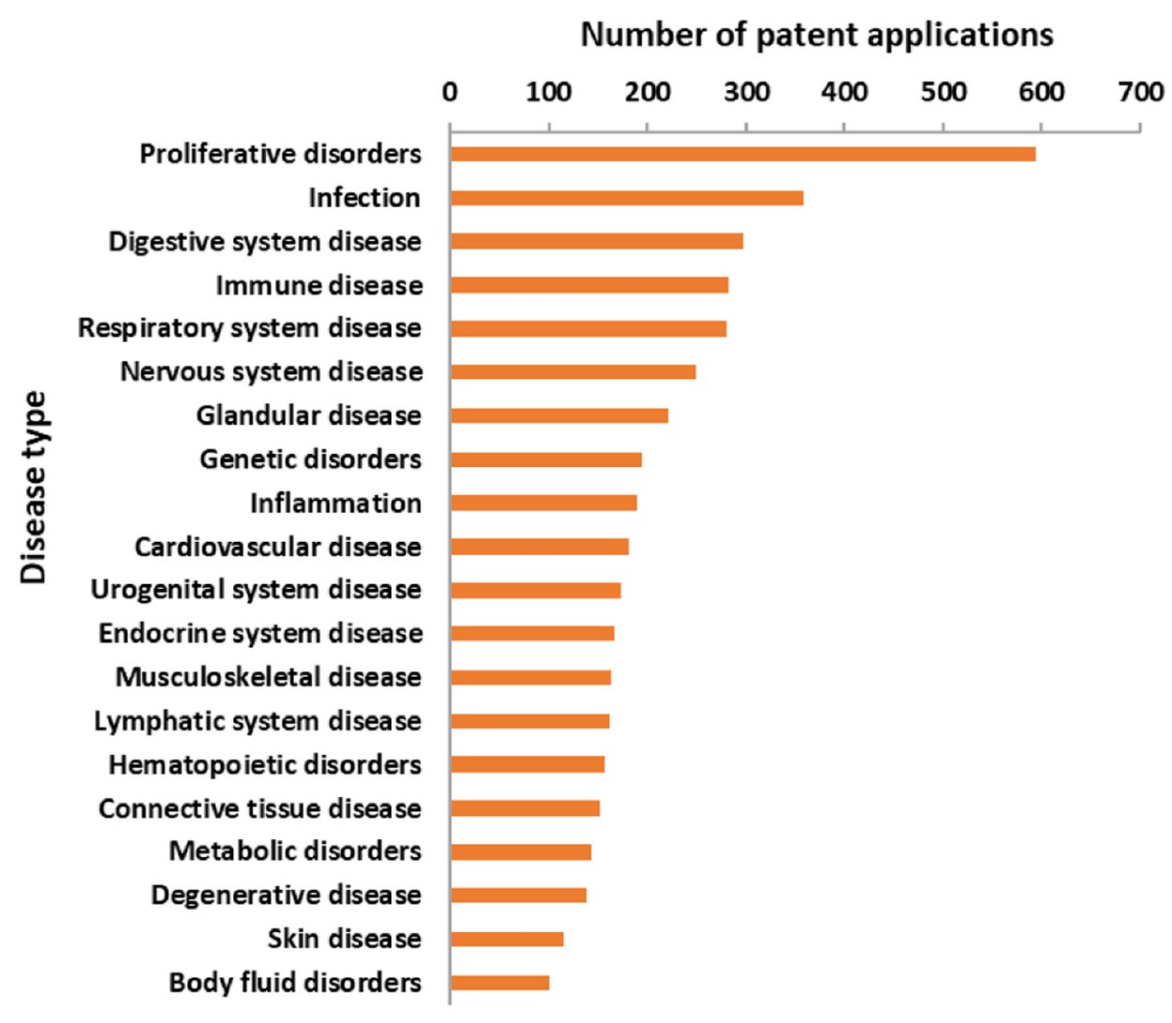
Distribution of patents for the top 20 primary diseases.

Diseases covered in mRNA patents include 69 primary disease classes, and the top 20 disease classes are shown in Figure 12. Proliferative disorders such as neoplasm, are claimed in the largest number of patents (600) followed by infectious diseases (358), indicating strong focus on application of mRNA medicines in these two areas. From the anatomical perspective, digestive system diseases, immune diseases, respiratory system diseases, nervous system diseases, and glandular diseases have been studies with relative high frequencies in patents. Disease classes such as genetic disorders and others are involved in fewer than 200 patent applications.

### Analysis of Proliferative Diseases Disclosed in Patents Related to mRNA Therapeutics and Vaccines

**Figure 13:**
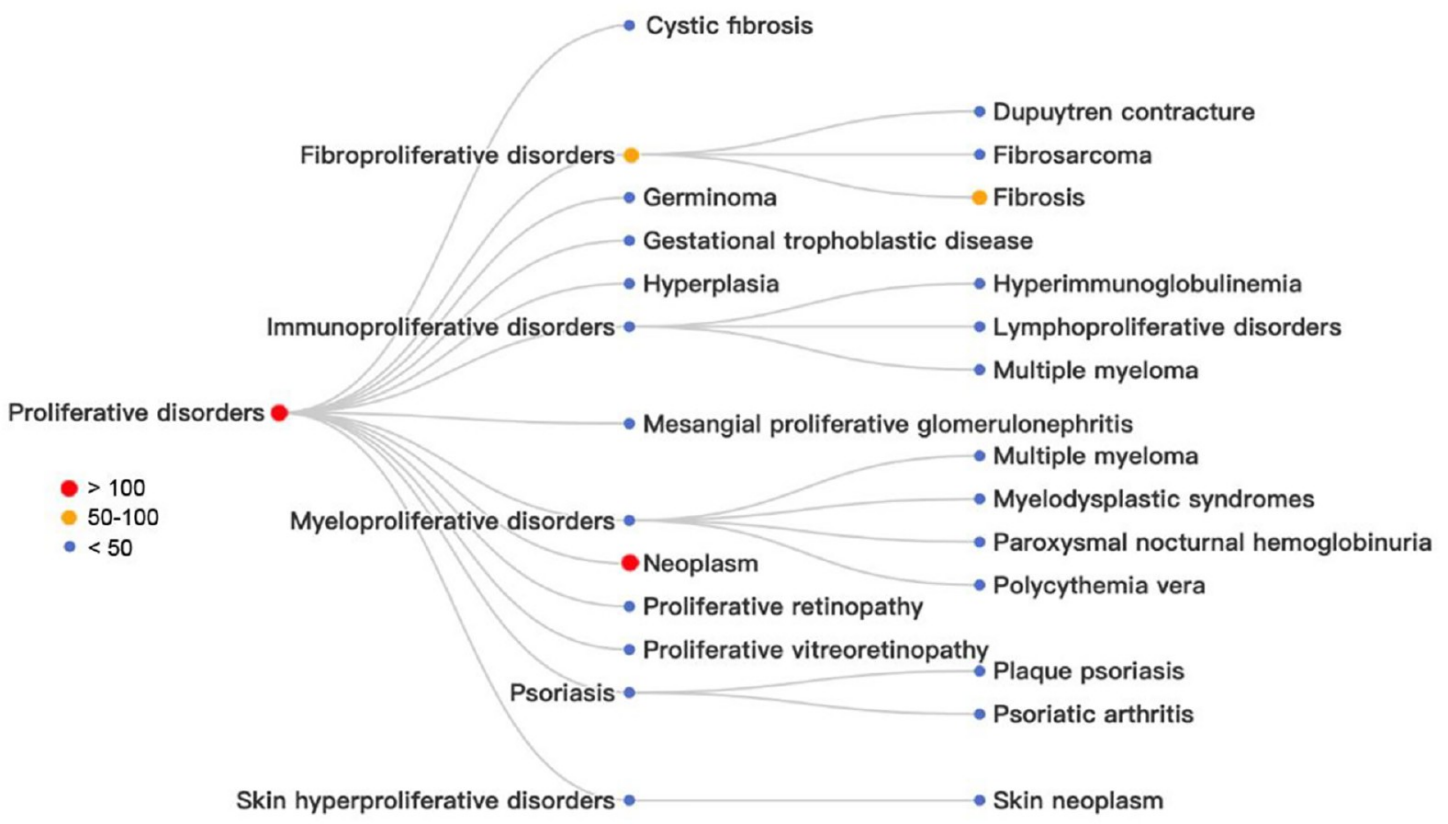
Analysis of proliferative diseases claimed by patents related to mRNA therapeutics and vaccines: red, more than 100 patents; orange, 50–100 patents; blue, less than 50 patents.

**Figure 14:**
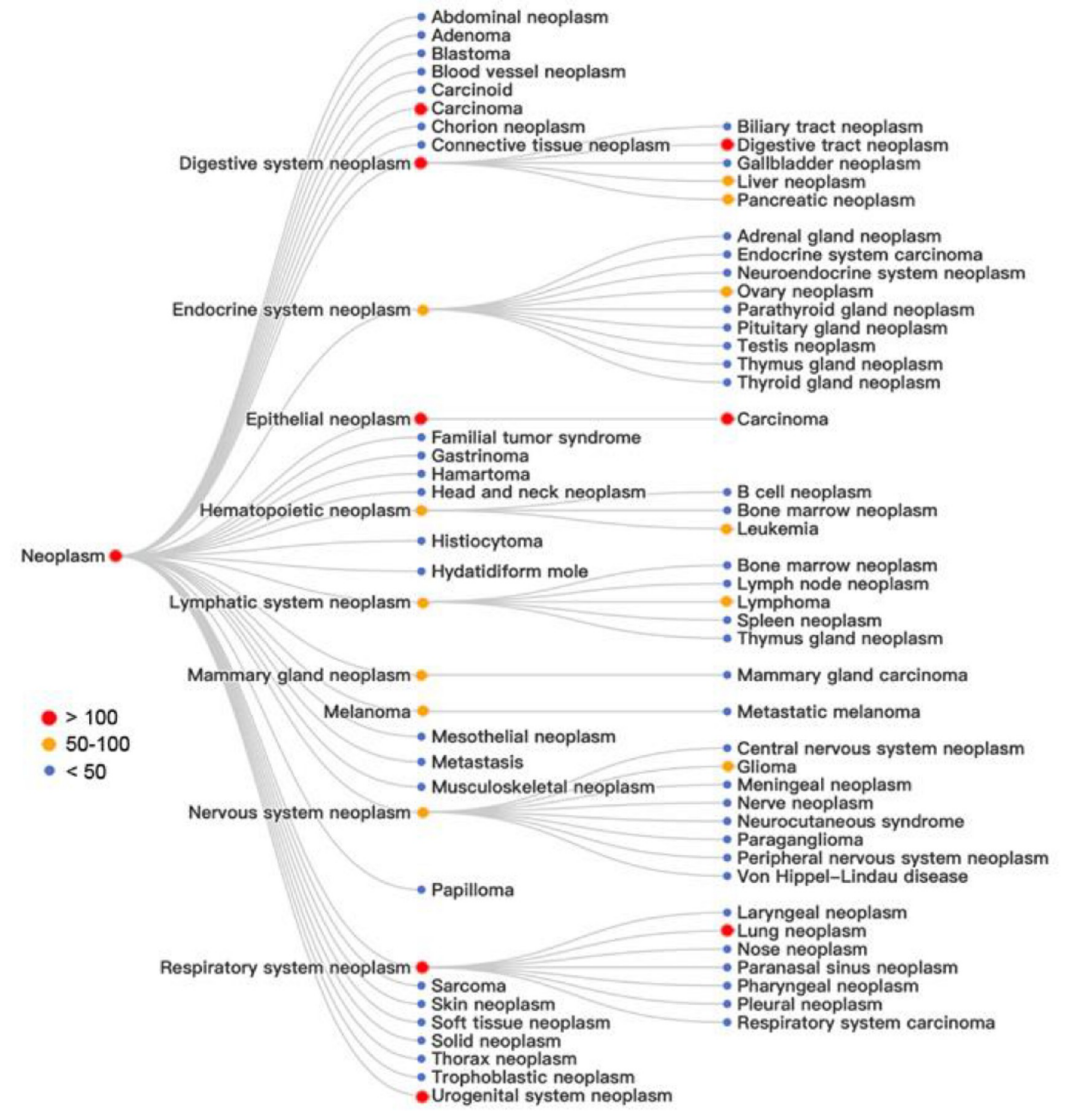
Analysis of key concepts in patents related to neoplasm: red, more than 100 patents; orange, 50–100 patents; blue, less than 50 patents.

Figure 13 shows classes of diseases claimed by patents related to mRNA therapeutics and vaccine development. The diseases are arranged hierarchically with the number range of patents involved indicated by dots in different colors. As shown in the figure, proliferative diseases such as neoplasm are the most investigated diseases in the R&D of mRNA therapeutics and vaccines. Within this broader class of diseases, neoplasm is the most explored for mRNA therapeutics and vaccines and appeared in 528 patents, indicating a strong interest in applying this new class of medicines to cancer prevention and treatment. As shown in Figures 13 and 14, among the more specific diseases, digestive system neoplasm, lung neoplasm, urogenital system neoplasm, and various forms of carcinoma (i.e., epithelial tissue-derived neoplasm) attracted the most attention, with more than 100 patents involved in each case.

## Patent Distribution among Infectious Diseases

**Figure 15:**
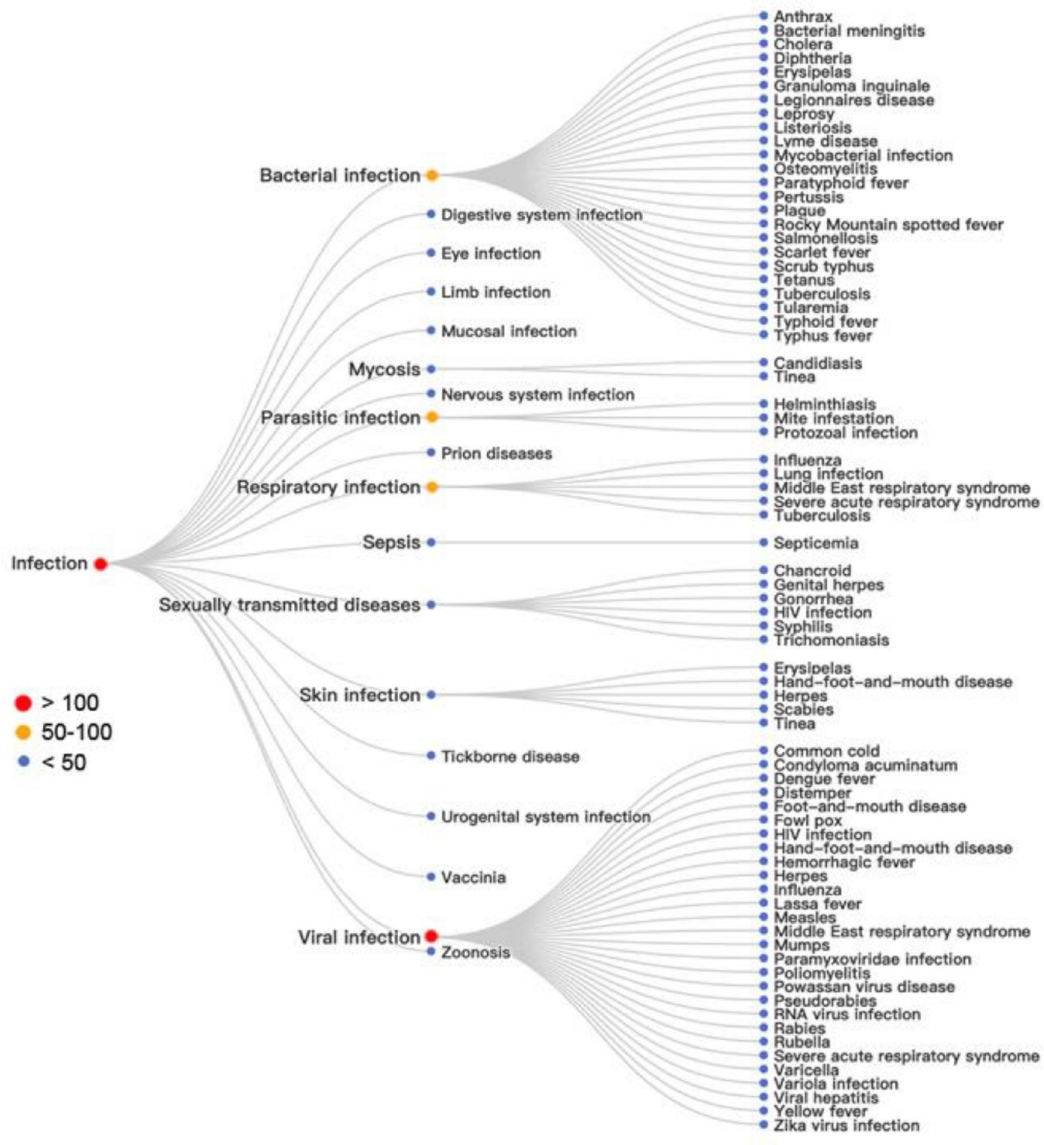
Analysis of infectious diseases claimed by patents related to mRNA therapeutics and vaccines: red, more than 100 patents; orange, 50–100 patents; blue, less than 50 patents.

As shown along the hierarchical tree of infectious diseases in Figure 15, there are 16 classes and over 60 specific diseases explored by patents on mRNA therapeutics or vaccines. Among those classes of diseases, viral infection has been most examined. Bacterial infections and parasitic infections were also of high concern. From the anatomical system perspective, respiratory system infection received more attention than other systems. Most of these patents are related to vaccine development against infectious diseases.

### Analysis of Immune Diseases Disclosed by Patents Related to mRNA Therapeutics and Vaccines

**Figure 16:**
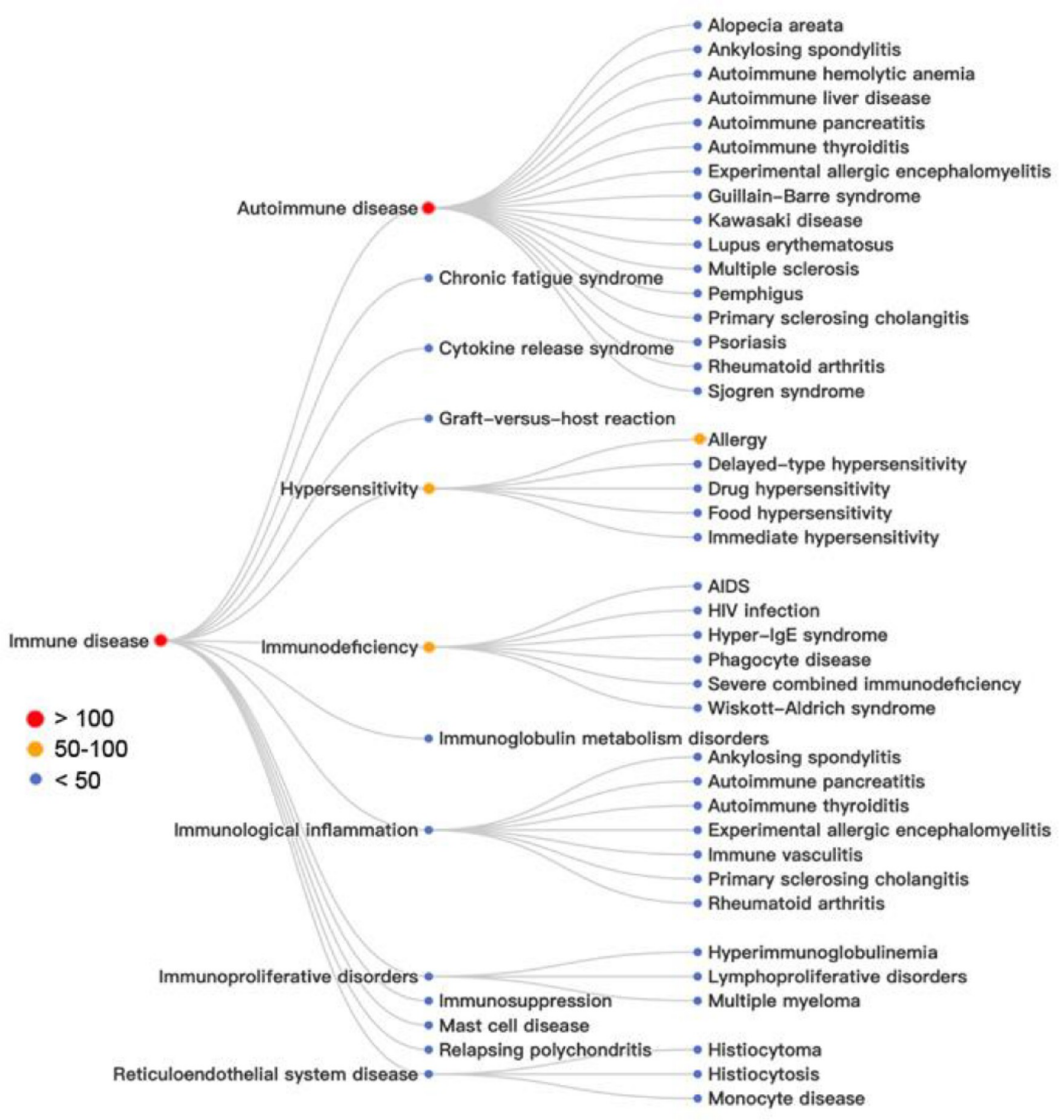
Analysis of key concepts in patents related to immune diseases: red, more than 100 patents; orange, 50–100 patents; blue, less than 50 patents.

Among various immune diseases examined by the mRNA therapeutic and vaccine patents, autoimmune diseases, followed by hypersensitivity (exaggerated response or over reaction to an antigen such as in the case of allergy) and immunodeficiency diseases, received the most attention as shown in Figure 16.

## mRNA Vaccines and Therapeutics in Clinical Trials

### Action Mechanism of mRNA Vaccines and Therapeutics

**Figure 17:**
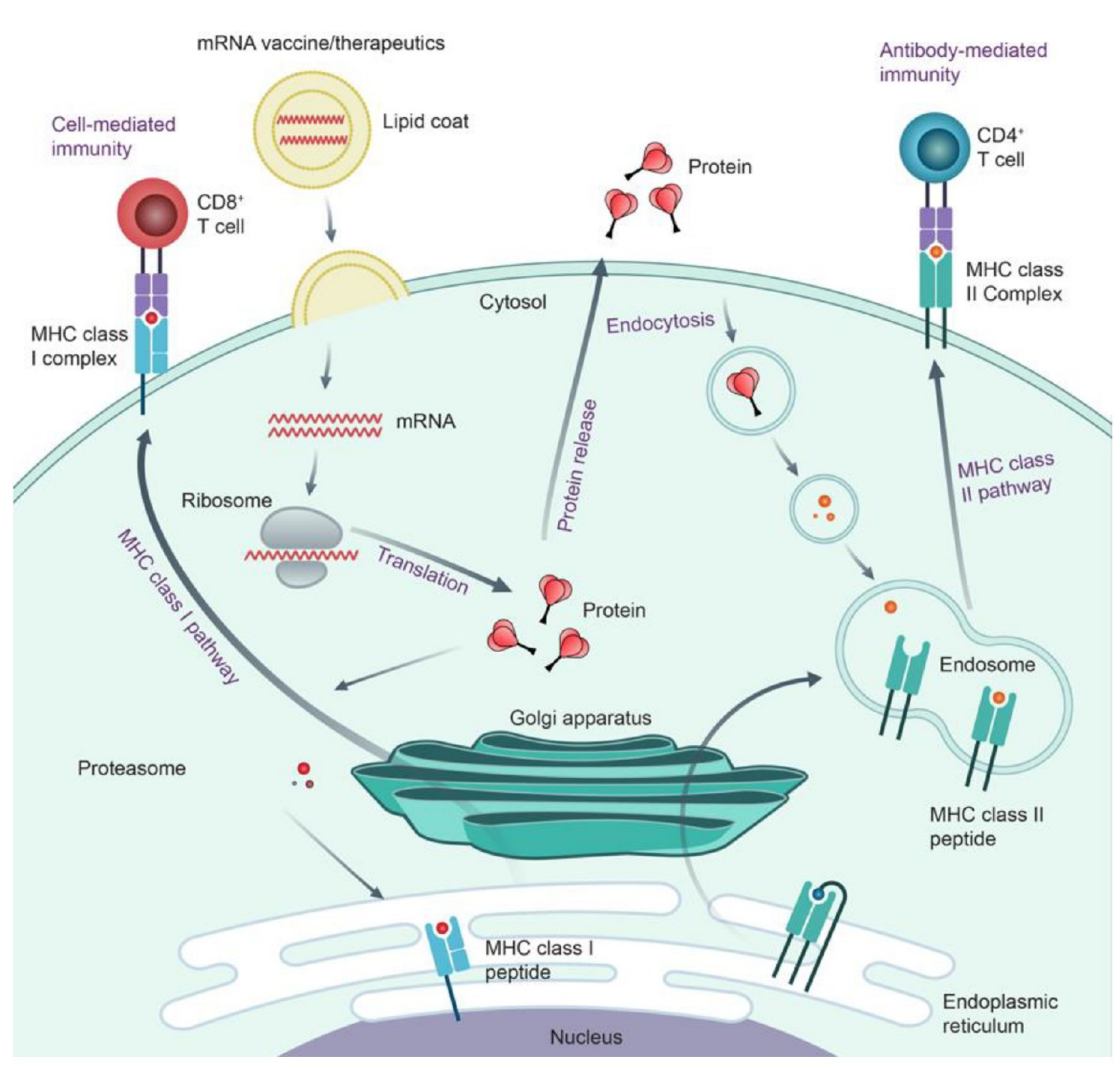
Mechanism of action of mRNAs vaccines and therapeutics (adapted and modified from ref (50)).

When the lipid nanoparticle (LNP) encapsulating mRNAs encoding targeted protein (antigenic protein) are administered in the body, LNP-mRNAs are engulfed by endocytosis and mRNAs are released to cytosol through endosomal escaping mechanism in antigen-presenting cells (no shown in the figure).^49^ Inside the ribosomes, a cellular machinery, proteins are translated based on the mRNAs. mRNA-encoded protein therapeutics use synthetic mRNAs that produce the desired proteins, such as antibodies, cytokines, and enzymes inside the human body. For vaccines, mimicking the viral infection process, intracellular produced antigens mainly elicit cell-mediated and antibody-mediated immunities. (Figure 17) First, the proteasome degrades antigenic proteins into peptide epitopes, which are transported into the endoplasmic reticulum and loaded onto major histocompatibility complex class I molecules (MHC I). The MHC I-peptide epitope complexes are presented on the surface of cells that bind to the T cell receptor to activate CD8+ T cells and kill infected or cancer cells (cell-mediated immunity). The antigenic proteins are transported via the Golgi apparatus and released to the outside of the cells. The secreted proteins are endocytosed by antigen-presenting cells, degraded, and loaded onto the MHC II peptide inside endosomes. The MHC II-peptide epitope complexes are presented on the surface of cells, which is recognized by CD4^+^ T cells facilitating B cells to make antigen-specific antibodies (antibody-mediated immunity).^50^

## mRNA Vaccines in Clinical Trials

Top companies for mRNA vaccine research include Moderna, BioNTech, Pfizer, and CureVac. Examination of diseases investigated by mRNA vaccines in clinical trials revealed that the vast majority (80%) of mRNA vaccines were designed for infectious diseases such as coronavirus infection, influenza, human immunodeficiency virus (HIV), rabies, and respiratory syncytial virus (RSV), among others. Other mRNA vaccines in the pipeline are being researched for various forms of cancers.

Table 10 lists mRNA vaccine candidates for infectious diseases currently in at least phase II trials. All mRNA vaccine candidates are developed for COVID-19 and encode the spike protein of SARS-CoV-2 or its receptor-binding domain. mRNA-1283 is a potential refrigerator-stable COVID-19 vaccine comprising mRNA encoding a SARS-CoV-2 spike protein N-terminal fragment and receptor-binding domain formulated in lipid nanoparticles. It is considered as the “next generation” vaccine candidate aiming for a pan-human coronavirus domain vaccine.

**Table 10:** mRNA Vaccine Candidates for Infectious Diseases in Phase 2 or More Advanced Clinical Trials^a^

**Vaccine name**	**CAS Registry Number**	**Disease indication**	**Antigen**	**Company**
BNT-162b2 (B.1.1.7 + B.1.617.2)	2883464-25-1	COVID-19	Prefusion stabilized S protein of SARS-CoV-2 B.1.1.7 and B.1.1.617.2 variants	BioNTech, Pfizer
BNT162b2 (B.1.351)	Pending	COVID-19	Prefusion stabilized S protein of SARS-CoV-2 B.1.351 variant	BioNTech,Pfizer
BNT 162b2 (B.1.1.529)	Pending	COVID-19	Prefusion stabilized S protein of SARS-CoV-2 B.1.1.529 variant	BioNTech, Pfizer
BNT-162b2 (WT/OMI BA.1)	Pending	COVID-19	Prefusion stabilized S protein of SARS-CoV-2 WT and BA.1 variant	BioNTech, Pfizer
BNT-162b5 (WT/OMI BA.2)	Pending	COVID-19	Prefusion stabilized S protein of SARS-CoV-2 WT and BA.2 variant	BioNTech, Pfizer
mRNA 1273	Pending	COVID-19	The full-length prefusion stabilized S protein	Moderna
mRNA 1273.211	2805221-47-8	COVID-19	Prefusion stabilized S protein of the SARS-CoV-2 and B.1.351 variant	Moderna
mRNA 1273.214	Pending	COVID-19	bivalent of SARS-CoV-2 spike protein from Beta and Delta variants	Moderna
mRNA 1273.351	2642373-67-7	COVID-19	the full-length prefusion stabilized S protein of the SARS-CoV-2 B.1.351 variant	Moderna
mRNA 1273.529	2763208-92-8	COVID-19	Prefusion stabilized S protein of the SARS-CoV-2 B.1.1.529 variant	Moderna
mRNA 1273.617	2882950-03-8	COVID-19	Prefusion stabilized S protein of the SARS-CoV-2 B.1.1.617.2 variant	Moderna
mRNA 1283	2696398-77-1	COVID-19	SARS-CoV-2 spike protein receptor-binding domain and N-terminal fragment	Moderna
mRNA 1283.529	Pending	COVID-19	Prefusion stabilized S protein of the SARS-CoV-2 B.1.1.529 variant	Moderna
mRNA 1283.211	2882951-80-4	COVID-19	Prefusion stabilized S protein of the SARS-CoV-2 B.1.351 variant	Moderna
LVRNA 009	Pending	COVID-19	SARS-CoV-2 spike protein	AIM Vaccine
ARCT 165	2714576-70-0	COVID-19	Self-Transcribing and Replicating mRNA encoding SARS-CoV-2 spike protein variants	Arcturus Therapeutics
ARCT 154	2698334-90-4	COVID-19	Self-Transcribing and Replicating mRNA encoding SARS-CoV-2 spike protein	Arcturus Therapeutics
ARCT 021	2541451-24-3	COVID-19	Self-Transcribing and Replicating mRNA encoding SARS-CoV-2 spike protein variants	Arcturus Therapeutics
BCD 250^b^	2756425-11-1	COVID-19	The receptor-binding domain of SARS-CoV-2 spike protein	Biocad
COVID-19 mRNA vaccine	Pending	COVID-19	SARS-CoV-2 spike protein	CanSino Biologics
SYS 6006	Pending	COVID-19	SARS-CoV-2 spike protein	CSPC Pharmaceutical
DS 5670	2749556-96-3	COVID-19	SARS-CoV-2 spike protein	Daiichi Sankyo
HDT 301	2437182-02-8	COVID-19	Self-amplifying RNA encoding SARS-CoV-2 spike protein	Emcure Pharmaceuticals
EG-COVID	Pending	COVID-19	SARS-CoV-2 spike protein	EyeGene
PTX-COVID19-B	2726459-47-6	COVID-19	SARS-CoV-2 spike protein	Providence Therapeutics
SW-BIC-213	2699076-70-3	COVID-19	The full-length SARS-CoV-2 spike protein	Stemirna Therapeutics
ABO1009-DP	Pending	COVID-19	SARS-CoV-2 omicron variant spike protein	Suzhou Abogen Biosciences
ARCoV	2543878-98-2	COVID-19	The receptor-binding domain of SARS-CoV-2 spike protein	Suzhou Abogen Biosciences
Awcorna	Pending	COVID-19	SARS-CoV-2 spike protein receptor-binding domain	Walvax Biotechnology
Comirnaty	2417899-77-3	COVID-19	The full-length prefusion stabilized S protein	BioNTech
Spikevax	2430046-03-8	COVID-19	The full-length prefusion stabilized S protein	Moderna
mRNA 1073	2760527-92-0	COVID-19 + influenza	Prefusion stabilized S protein of SARS-CoV-2 and hemagglutinin	Moderna
mRNA 1345	2766353-31-3	Respiratory syncytial virus infection	RSV prefusion stabilized F glycoprotein	Moderna
BNT 161	2760529-48-2	Influenza	Hemagglutinin from H1N1 and B/Yamagata influenza strains	BioNTech
mRNA 1010	2760527-87-3	Influenza	Hemagglutinin from four seasonal influenza strains	Moderna
mRNA 1020	2760527-90-8	Influenza	Hemagglutinin and neuraminidase antigens	Moderna
mRNA 1030	2760527-91-9	Influenza	Hemagglutinin and neuraminidase antigens	Moderna
MRT 5407	2900351-99-5	Influenza	Quadrivalent influenza vaccine	Sanofi
mRNA 1893	2882947-97-7	Zika virus infection	Structural proteins of Zika virus	Moderna
mRNA 1325	2882946-55-4	Zika virus infection	Structural proteins of Zika virus	Moderna
mRNA 1647	2882946-59-8	CMV infection	Six mRNAs coding for pentamer viral antigen and glycoprotein B of CMV	Moderna

a Data from the CAS Content Collection, Clinicaltrials.gov, and Pharmaprojects.

b Vaccine no longer in development.

In addition to mRNA vaccines for SARS-CoV-2 infection, several mRNA vaccine candidates for infection by other viruses such as RSV, influenza virus, Zika virus, and cytomegalovirus (CMV) have entered clinical trials. In January 2023, Moderna announced that RSV vaccine, mRNA-1345, demonstrated vaccine efficacy of 83.7% at preventing symptoms in older adults in randomized phase III trial.^51^ Moderna plans to submit mRNA-1345 for regulatory approval in the first half of 2023.^52^

Two research groups examined levels of neutralizing antibodies and differences in CD4+ or CD8+ T cell responses induced by monovalent and bivalent COVID-19 booster vaccines for protecting against omicron variants. Neither group observed superior immune responses to bivalent booster vaccines compared to monovalent vaccines. Most of neutralizing antibodies elicited by vaccines targeting newer variants still recognize only the original virus because of “immune imprinting” in which the body repeats its immune response to the first variant encountered.^53,54^ However, fine-tuning the dosage of booster vaccines might increase their efficacy of protection again immune-escape COVID-19 variants.^55^

The success of COVID-19 mRNA vaccine has revealed the application potential of mRNA platform not only for expansion to other infectious diseases but also for cancers (Table 11), especially as therapeutic vaccines The clinical study of mRNA vaccines has shown good efficacy in the treatment of melanoma, non-small-cell lung cancer (NSCLC), and prostate cancer. Ref. Autogene cevumeran (RO 7198457, BNT122), jointly developed by BioNTech and Genentech, is a mRNA-based individualized neoantigen specific immunotherapy (iNeST). It encodes up to 20 neoepitopes defined by the patient’s tumor-specific mutations delivered in an RNA-lipoplex formulation. A combination of intravenously administered autogene cevumeran combined with anti-PD-L1 immune checkpoint inhibitor atezolizumab is conducted in patients with locally advanced or metastatic solid tumors has entered a first-in-human phase I study. It has induced strong CD4+ and CD8+ T cell immunity against neoantigens. Randomized phase II studies of autogene cevumeran for patients with melanoma in combination with pembrolizumab, for individuals with non-small-cell lung cancer (NSCLC) in combination with atezolizumab, and for individuals with colorectal cancer (CRC) are currently ongoing.

**Table 11:** mRNA Vaccines in Phase 2 or More Advanced Clinical Trials for Cancers^a^

**Vaccine name**	**CAS Registry Number**	**Disease indication**	**Antigen**	**Company**
Autogene cevumeran	2365453-34-3	Melanoma; colorectal cancer	Patient-specific neoantigens	BioNTech
mRNA 4157	2741858-84-2	Melanoma	Up to 34 neoantigens	Moderna
mRNA 4359	2900354-08-5	Melanoma; non-small-cell lung carcinoma	IDO and PD-L1	Moderna
BNT 111	2755828-88-5	Melanoma	Mix of four melanoma-associated antigens	BioNTech
BNT 112	2900354-09-6	Prostate cancer	Mix of five prostate cancer-specific antigens	BioNTech
BNT 113	2882951-85-9	PV16+ head-and-neck squamous carcinoma	HPV16-derived tumor antigens (oncoprotein E6 and E7)	BioNTech
CV 9202	1665299-76-2	Non-small-cell lung cancer	NY-ESO-1, MAGE C1, MAGE C2, TPBG (5T4), survivin, MUC1	CureVac
CV 9103	2882951-83-7	Prostate cancer	Mix of four prostate cancer-associated antigens	CureVac
SW 1115C3	2882951-82-6	Non-small-cell lung cancer; esophageal cancer	Patient-specific neoantigens	Stemirna Therapeutics
Rocapuldencel T; AGS 003	2396421-01-3	Non-small-cell lung cancer; lung cancer; bladder and renal cancer	Autologous tumor antigen and CD40L-loaded dendritic cell immunotherapy	Argos Therapeutics

a Data from the CAS Content Collection, Clinicaltrials.gov, and Pharmaprojects.

BNT111 developed with the BioNTech’s FixVac platform encodes four tumor-associated antigens (TAAs), the cancer-testis antigen NYESO-1, the human melanoma-associated antigen A3 (MAGE-A3), tyrosinase, and putative tyrosine-protein phosphatase (TPTE) and is encapsulated in an RNA-lipoplex formulation. It has entered phase II clinical trials to treat advanced melanoma and has gained FDA fast track designation in 2021. A report from a phase II trial showed that the use of BNT111 alone or in combination with PD-1 antibody can induce tumor antigen-specific CD4+ and CD8+ T cell immune responses.

CV9201, developed by CureVac, is an RNA-based therapeutic vaccine encoding five NSCLC antigens. The first-in-human, multicenter, phase I/IIa study was conducted in 7 patients with locally advanced NSCLC and 39 patients with metastatic NSCLC. The result demonstrated that specific immune responses against at least one antigen were detected in 63% of patients after treatment and the frequency of activated IgD+ CD38hi B cells increased by more than 2-fold in 60% of evaluated patients.^56^

Moderna’s personalized mRNA cancer vaccine, mRNA-4157, encodes 34 unique neoantigen genes that may stimulate specific T cell responses. Phase I trials showed that this vaccine is safe and tolerable in monotherapy or in combination with pembrolizumab.^57^ In December 2022, Moderna and Merck announced that mRNA-4157 in combination with anti-PD-1 antibody, pembrolizumab reduced the risk of recurrence or death by 44% in patients with stage III/IV melanoma compared with pembrolizumab monotherapy based on their randomized phase IIb trial.^58^

## mRNA Therapeutics in Clinical Trials

Top companies for mRNA therapeutic research include BioNTech, Moderna, Arcturus Therapeutics, AstraZeneca, and Sanofi. mRNA therapeutics have a broad range of targeted diseases. Consistent with the patent-disease analysis data above, mRNA therapeutics are being developed largely for cancers, followed by metabolic, cardiovascular, infectious, immunological, and respiratory diseases.

mRNA therapeutic products currently in clinical trials are examined in Table 12 to reveal a landscape view of the current progress in mRNA therapeutics in the clinical development pipeline. A select few are also examined in further detail below to showcase the variety of mRNA therapeutics, their mechanism of actions, and their targeted disease indications.

**Table 12:** mRNA Therapeutic Products in Clinical Trials^62^^a^

**mRNA drug name**	**CAS Registry Number**	**Disease indications**	**Description**	**Company**
A-001; TriMix-MEL; ECL-006; E011-MEL	2877674-59-2	Melanoma	A mixture of three mRNAs encoding constitutively activated CLT4, CD40L, and TLR4 plus mRNAs for five melanoma-associated antigens (tyrosinase, gp100, MAGE-A3, MAGE-C2, and PRAME), which activate key immune cells against cancer	eTheRNA Immunotherapies
ARCT-810; LUNAR-OTC	2877704-48-6	Ornithine trans-carbamylase deficiency	mRNA encoding ornithine transcarbamylase formulated in a lipid nanoparticle to correct the enzyme deficiency	Arcturus Therapeutics
AZD-8601	2603440-18-0	Heart failure and ischemic cardiovascular diseases	mRNA encoding vascular endothelial growth factor A to stimulate new vascular blood vessel formation and repair as well as regenerate heart cells	AstraZeneca
BD-111	2901016-63-3	Herpetic viral keratitis	Viral-like particle drug delivery system used to transduce cas9 mRNA that directly targets and cuts the viral genome of herpes simplex virus 1 to effectively remove the virus	BD Gene
BNT-141	2877707-22-5	Solid tumors	mRNA encoding a monoclonal antibody targeting claudin 18, a protein commonly expressed in multiple cancers	BioNTech
BNT-142	2877707-34-9	Solid tumors	mRNA encoding a bispecific antibody targeting CD3, a protein involved in activation of certain types of T cells, and claudin 6 (CLDN6), a protein highly expressed in certain cancers	BioNTech
BNT-151	2877709-82-3	Solid tumors	A nucleoside-modified, cationic lipoplexes-loaded mRNA encoding an interleukin-2 (IL-2) variant to stimulate anti-cancer T cells	BioNTech
BNT-152	2877709-92-5	Solid tumors	A nucleoside-modified mRNA encoding interleukin-7 to stimulate anti-cancer T cells	BioNTech
BNT-153	2877709-93-6	Solid tumors	A nucleoside-modified mRNA encoding interleukin 2 (IL-2) to stimulate anti-cancer T cells	BioNTech
LioCynx-M004; Lion TCR	2901015-92-5	Hepatitis B virus-related hepatocellular carcinoma	A genetically modified autologous cell therapy derived from T cells transfected with mRNA encoding to express a T-cell receptor that recognizes the hepatitis B surface antigen on the surface of HBV-related cancer cells	Lion TCR
MEDI-1191	2877712-03-1	Solid tumors	LNP-encapsulated mRNA encoding IL-12 to increase intratumor production of IL-2 via intratumoral injection	Moderna
mRNA-2752	2878461-50-6	Solid tumors	Lipid nanoparticle-encapsulated mRNAs encoding human T cell co-stimulator, OX40L, and proinflammatory cytokines, IL-23 and IL36γ, for intratumoral injection	Moderna
mRNA-3705	2878470-78-9	Methylmalonic acidemia	Lipid nanoparticle-encapsulated mRNA encoding the mitochondrial enzyme methyl-CoA mutase that is deficient in methylmalonic acidemia	Moderna
mRNA-3745	2878574-58-2	Glycogen storage disease type 1a	Lipid nanoparticle-encapsulated mRNA encoding glucose 6-phosphatase to restore the deficient enzyme responsible for converting glycogen into glucose for treatment of type 1a glycogen storage disease	Moderna
mRNA-3927	2878577-32-1	Propionic acidemia	Lipid nanoparticle-encapsulated mRNAs encoding propionyl-CoA carboxylase α subunit and β subunit to restore the deficient enzyme and reduce toxic buildup of some substances in propionic acidemia	Moderna
mRNA-6231^b^	2878577-39-8	Autoimmune diseases	Lipid nanoparticle-encapsulated modified mRNA encoding a mutated form of human IL-2 fused to human serum albumin to increase its half-life to restore IL-2- and T-cell-mediated immune homeostasis	Moderna
MRT-5005^b^	2328142-67-0	Cystic fibrosis	Inhalable form of engineered mRNA variant encoding fully functional cystic fibrosis transmembrane conductance regulator protein for restoring lung function in cystic fibrosis; Phase I/II clinical trial (NCT03375047) showed no improvement in lung function but did discover that repeated inhaled doses of mRNA are safe^63^	Translate Bio (acquired by Sanofi)
SAR-441000	2879301-17-2	Solid tumors	Mixture of four mRNAs encoding IL-2 single chain, IL-15 fused to the sushi domain of IL-15Rα, GM-CSF, and interferon α2b, which have been reported as mediators of tumor regression	Sanofi, BioNTech
SQZ-eAPC-HPV	2879306-51-9	HPV and solid tumors	mRNA-based cell therapeutic agent that delivers five mRNAs for HPV16 protein antigens and immune-stimulating proteins, including CD86 and membrane bound IL-2 and IL-12, into four different types of engineered immune cells (monocytes, T-cells, B-cells, and NK cells) of cancer patients in a single step	SQZ Biotechnologies
UX053; LUNAR-GSDIII	2901003-30-1	Glycogen storage disease type III	Lipid nanoparticle-encapsulated mRNA therapeutic encoding the glycogen debranching enzyme; replacing the defective AGL gene product allows cells to break down glycogen using normal pathways	Ultragenyx, Arcturus Therapeutics
Verve-101	2894841-30-4	Heterozygous familial hypercholesterolemia	mRNA-based lipid nanoparticle therapeutic that targets the liver and base-editing in the PCSK9 gene to disrupt PCSK9 protein production to lower LDL cholesterol and treat cardiovascular disease	Verve Therapeutics

a Data from the CAS Content Collection, Clinicaltrials.gov, and Pharmaprojects.

b Drug no longer in development.

Arcturus Therapeutics developed and is currently evaluating a mRNA therapeutic for the treatment of ornithine transcarbamylase (OTC) deficiency that currently has no FDA-approved treatments. The urea cycle enzyme OTC helps remove ammonia from liver cells, and a deficiency leads to high ammonia levels. Utilizing their lipid-mediated nucleic acid delivery system (LUNAR), Arcturus is currently researching LUNAR-OTC in a phase II clinical trial (NCT05526066) to evaluate its safety and tolerability in participants with OTC deficiency.^59^

AZD8601 is a mRNA therapeutic developed through a collaboration between AstraZeneca and Moderna. It encodes for VEGF-A, a paracrine factor important for new blood vessel formation and progenitor cell division that contributes to repair and regeneration of the heart. A phase II clinical trial (NCT03370887) examined the safety, tolerability, and exploratory efficacy of an intra-myocardium AZD8601 injection in patients with moderately impaired systolic function undergoing coronary artery bypass grafting surgery. Trial results revealed no serious side effects and a rising trend in efficacy for the treatment group.^60^

SQZ Biotechnologies, in collaboration with Roche, has developed a mRNA-based cell therapeutic called SQZ-eAPC-HPV. SQZ-eAPC-HPV delivers five mRNAs for HPV16 protein antigens and immune-stimulating proteins, including CD86 and membrane-bound IL-2 and IL-12, into four different types of engineered immune cells (monocytes, T-cells, B-cells, and NK cells). A phase I/II clinical trial (NCT05357898) is currently recruiting to assess safety and tolerability, antitumor activity, and immunogenic and pharmacodynamic effects of SQZ-eAPC-HPV as monotherapy and in combination with pembrolizumab in patients with recurrent, locally advanced, or metastatic HPV16+ solid tumors.

BioNTech developed BNT141, a mRNA that encodes secreted IgG antibodies targeting claudin 18.2, a protein commonly expressed on certain solid tumors. It is being researched in phase I/II clinical trial number NCT04683939, looking at its safety and pharmacokinetics in patients with Claudin 18.2-positive solid tumors.^61^

## Methods

This study used the CAS Content Collection as the primary data source, which aggregates and connects key scientific details disclosed in more than 50,000 journals and patents from 64 issuing authorities, as well as many other sources to cover the scope of science from chemistry and life sciences to engineering, materials science, and agriculture. A search query that searched for words or phrases in titles, abstracts, keywords, CAS index terms, and substances was developed to extract all the scientific journal articles and patents related to mRNA vaccines and therapeutics. The resulting patents were then intellectually reviewed to 1) remove false drops and 2) classify each document into one or more of the following classes: a) vaccines, b) therapeutics, and/or c) delivery system as well as mRNA modification methods. During this review process, the likely intriguing/notable/highly influential patents were also tagged for further analyses.

Once such reviews were done, the answer set containing over 2,000 patents and more than 9,000 journal articles was further analyzed for patents and journal articles separately for the categories of mRNA vaccines and mRNA therapeutics. Specific analyses included the trends over time; landscape analyses including country distribution, patent office distribution, flow of patents from applicant home countries to patent offices, and most influential organizations; and topic cluster analyses. Most notable patents, diseases involved, and specific mRNA therapeutics and vaccines in clinical trials were also examined based on both CAS-licensed data and publicly available information. The analysis tools include Thomson Data Analyze (DDA), VOSviewer, OmniGraphSketcher, and ECharts.

At the document level, this report focused more on patents than journal articles. A group of patent applications covering the same or similar technical content is called a patent family. Patents for the same technological invention may be filed in multiple countries or regions, and the CAS database maintains these related applications as one record for indexing; this record is referred to as the basic patent for most analyses in this report. Individual patent applications from the same patent family may be applied for in different countries and/or patent offices. This properly represents the distribution of patent applications filed by applicants’ countries (i.e., applicants from different countries or organizations); individual patent applications from the same family are counted separately for geographic distribution and patent flow analysis. The country of origin of a patented technology is determined based on the country location of the patent assignee (or applicant/inventor). The country analysis in this report is based on the country location of the patent applicant for determination of the country of origin of the technology or the actual country owning the technology.

## Outlook and Perspectives

The remarkable success of mRNA vaccines against COVID-19 has strongly motivated interest in mRNA as a way of delivering therapeutic proteins. However, a variety of challenges remain to be resolved before mRNA can be verified as a common therapeutic modality with wide-ranging relevance to both rare and common diseases. The challenges in developing better mRNA formulations are as follows:

*• Enhancing specific protein expression: Elaboration of the mRNA cargo to augment the time and amount of protein production in vivo, including advances in the design of the primary chemical structure of the mRNA, novel forms of circular and self-amplifying mRNA, and improved purification strategies.* The most critical advances in mRNA vaccines and therapeutics are due to the discovery that the insertion of chemically modified nucleosides, specifically in uridine moieties, can significantly increase protein expression. So far, over 130 chemical modifications of RNA have been described, and the properties and effects of multiple other RNA chemical modifications remain to be explored.^20,64^ All parts of the mRNA—the cap, 5′ and 3′ regions, open reading frame, and polyadenylated tail—can be optimized to augment protein expression.^65^ In addition to the amount of protein expression, a crucial hurdle of mRNA therapeutics for chronic diseases is the relatively short time period of protein production, which requires repetitive administrations. Self-amplifying mRNAs (saRNAs) make use of the self-replication of an RNA alphavirus, which can amplify RNA transcripts in the cytoplasm.^66,67^ Circular mRNAs (circRNAs) provide another approach for extending the duration of protein production. The circular structure shields circRNA from exonuclease attacks, which prolongs the RNA lifespan, thus raising the total protein yield.^68^

• *Improving mRNA packaging and delivery systems, including ionizable LNPs, peptide-based nanoparticles, cells, and cell-based extracellular vesicles.* The rapid clinical implementation of mRNA-based vaccines and therapies has become possible due to the development of efficient delivery vehicles to protect and transport the highly unstable mRNAs. Various smart delivery systems have been invented to improve key features such as circulation time in blood stream, biodistribution, cargo loading, and release.

Lipid-based nanoparticles are currently the most widely studied and clinically advanced vehicles for mRNA delivery.^47,69−71^ The most extensively used are cationic and ionizable LNPs.^72^ Although cationic lipid-based nanoparticles have shown promise in certain therapies, their possible cytotoxicity^73^ and relatively short circulation time^74^ have somehow impeded their clinical application. To deal with these issues, a variety of novel lipid–PEG conjugates and ionizable lipids have been synthesized and tested. Ionizable lipids remain neutral at physiological pH, which reduces their toxicity and increases to some degree their circulation half-life.^75^ Furthermore, the protonation of ionizable lipids at acidic pH not only allows successful condensation and encapsulation of mRNAs, but also allows the escape of mRNAs from the acidic endosomes.^70,76^ The PEG shell set up by PEGylated lipids considerably prolongs the circulation half-life of the LNPs and reduces their aggregation; it also cut back unfavorable interactions with serum proteins.^77^

Another promising approach uses extracellular vesicles and especially their smallest version, the exosomes, as a delivery vehicle.^48^ Exosomes are nanosized vesicles enclosed by a lipid bilayer membrane. They are produced by most eukaryotic cells and subsequently released in the extracellular space, providing means of efficient intercellular communication and signaling, including transport of bioactive molecules such as proteins, nucleic acids, and metabolites between cells and across biological barriers.^78,79^ As natural delivery vehicles, exosomes possess multiple benefits, such as biocompatibility and low immunogenicity. It has been reported that exosomes originated from various cell types do not provoke toxicity and are well-tolerated after repetitive dosing.^80^ Thus, they can be viewed as promising mRNA delivery systems in cases in which repeated dosing is required.

Peptides represent other versatile, biocompatible, and targetable RNA carriers. From this class, cell-penetrating peptides (CPPs) that can penetrate the cellular membrane and transfer to the cytoplasm are used most frequently for RNA delivery by forming a variety of nanocomplexes depending on the formulation settings and the properties of the used CPPs and RNAs.^81,82^

A further novel biological alternative to lipid nanoparticle carriers is the use of cell-based delivery systems, exploiting the cellular paracrine communication ability to directly deliver proteins synthesized by mRNA brought into cells ex vivo. It provides certain advantages, such as biocompatibility, lack of toxicity, and extended half-life in circulation. Various modifications are feasible by introducing mRNA into cells such as immune cells, blood cells, and others.^83−85^ Recently, a bacteria-mediated vehicle for oral mRNA vaccine delivery has been also developed. This multiple-target vaccine successfully explored engineered Salmonella-based vector to deliver mRNA vaccine against Delta and Omicron variants of COVID-19.^86,87^

• *Targeting mRNA therapeutics to certain tissues and engineering of delivery systems with tissue-specific affinity.* Fulfilling the potential of mRNA therapeutics requires proper targeting. Liver is the largest internal organ in human body, performing vital roles in various physiological activities, thus large amount of disease targets potentially receptive to mRNA therapies reside within the liver hepatocytes. A promising liver-targeted N-acetylgalactosamine (GalNAc) ligand has been assessed in various trials for enhancing the cellular uptake and tissue specific delivery of mRNAs.^88^ GalNAc-based delivery relies on the ability of the hepatocytes to express the asialoglycoprotein receptor, a high-capacity, rapidly internalizing receptor that binds glycoproteins via receptor-mediated endocytosis. In recent years, encouraging progress has been made in the field of GalNAc conjugates, underscoring the value of targeting moieties.^89,90^ The conjugation of GalNAc moieties to oligonucleotides represents a promising and safe approach for liver-targeted delivery of nucleic acid therapeutics.

• *Selective delivery of mRNA therapeutics to organs other than liver requires specifically developed packaging and delivery systems with appropriate affinity.* The recent efforts on developing better delivery systems and/or administration routes for this purpose have produced some exciting results. For example, Daniel Siegwart’s team has recently developed a method of Selective ORgan Targeting (SORT) that uses various engineered lipid nanoparticles to selectively deliver mRNA to extrahepatic organs.^91^ Qiaobing Xu’s team has identified an endogenously lymph node-directing lipid nanoparticle system.^92^ Kathryn A. Whitehead’s team devised a strategy of delivering cationic helper lipid-containing lipid nanoparticles by intraperitoneal administration to deliver mRNA to pancreas, especially the insulin-producing cells.^93^ Gaurav Sahay’s lab has developed a LNP system with increased polyethylene glycol (PEG) and inclusion of a cholesterol analog, β-sitolsterol to optimize the delivery of the nebulized, LNP-based mRNA for fibrosis transmembrane conductance regulator (CFTR) protein as potential inhalable LNP-based mRNA therapies.^94^

• *Strategies for allowing repeated dosing for the treatment of chronic conditions.* The development of mRNA therapeutics raises further challenges as compared to those with mRNA vaccines. While immunization requires only a small amount of protein production, as the immune system will amplify the antigenic signal, mRNA therapeutics require much higher level of protein to reach a therapeutic level. The tissue bioavailability, half-life in circulation, and carrier efficiency to deliver to the targeted tissue remain challenging. Another major difficulty is the repeated dosing, which is typically required in the treatment of chronic diseases and which activates innate immunity in the long run, with a resultant reduction of therapeutic protein expression.^65^ Regardless of these hurdles, however, a variety of emerging technologies are currently under development with the effort to deal with them.^26,95−97^

Despite these challenges, impressive progress has been made in the research and development of mRNA therapeutics and vaccines. Most recently, Luis A. Rojas et al. have reported the promising result of a Phase 1 clinical trial in which personalized uridine mRNA neoantigen vaccines induced substantial T cell activity in postsurgery patients with pancreatic ductal adenocarcinoma.^98^ The remarkable success of the mRNA vaccines for COVID-19, along with recent expansion of this technology into treatment of most lethal cancers, has validated the great promise of this new class of medications and significantly enhanced the chance to witness their extensive applications in the near future.

## Supporting Information Available

SI-1, trend over time for patents related to mRNA therapeutics; SI-2, trend over time for patents related to mRNA vaccines; SI-3, trend over time for patents on mRNA delivery systems; SI-4, trend over time for patents related to mRNA modification; SI-5, analysis of research topics in mRNA therapeutics patents; SI-6, analysis of research topics in mRNA vaccine patents; SI-7, analysis of research topics in patents related to mRNA modification; and SI-8, top countries and organizations in the field of mRNA modification (PDF)
