# Supplementary material for: Messenger RNA-Based Therapeutics and Vaccines: What’s beyond COVID-19?
Source: ACS Pharmacol Transl Sci. 2023 Jul 3;6(7):943–69. doi: 10.1021/acsptsci.3c00047 (PMC10353067; doi:10.1021/acsptsci.3c00047)
Supplement: Supplementary file 1 — pt3c00047_si_001.pdf [file pt3c00047_si_001.pdf]

Supplemental Information

Messenger RNA (mRNA)-based therapeutics and vaccines: What's beyond COVID-19

Dongqiao Li[†1], Cynthia Liu[†2], Yingzhu Li[2], Rumiana Tenchov[2], Janet M. Sasso[2], Di Zhang[1], Dan Li[1], Lixue Zou[1], Xuezhao Wang[*1], and Qiongqiong Zhou[*2]

[1] Information Center, National Science Library, Chinese Academy of Science, Haidan District, Beijing 100190

[2] CAS, a division of the American Chemical Society
2540 Olentangy River Rd, Columbus, OH 43202, USA

[†]Authors D.L. and C.L. contributed equally to this paper

*Corresponding author: wangxz@mail.las.ac.cn

*Corresponding author: qzhou@cas.org

Supplemental Information

SI-1. Trend over time for patents related to mRNA therapeutics

The annual number of global patents in the field of mRNA therapeutics increased steadily from 7 in 2000 to 180 in 2021 although the increase has been more significant for the past several years (Figure S1). Since 2000, fewer patents have been filed in this field. The At the end of 2019, probably catalyzed by the COVID pandemic, technology patents rose in the field of mRNA therapeutics worldwide, and the number of s each year increased significantly.

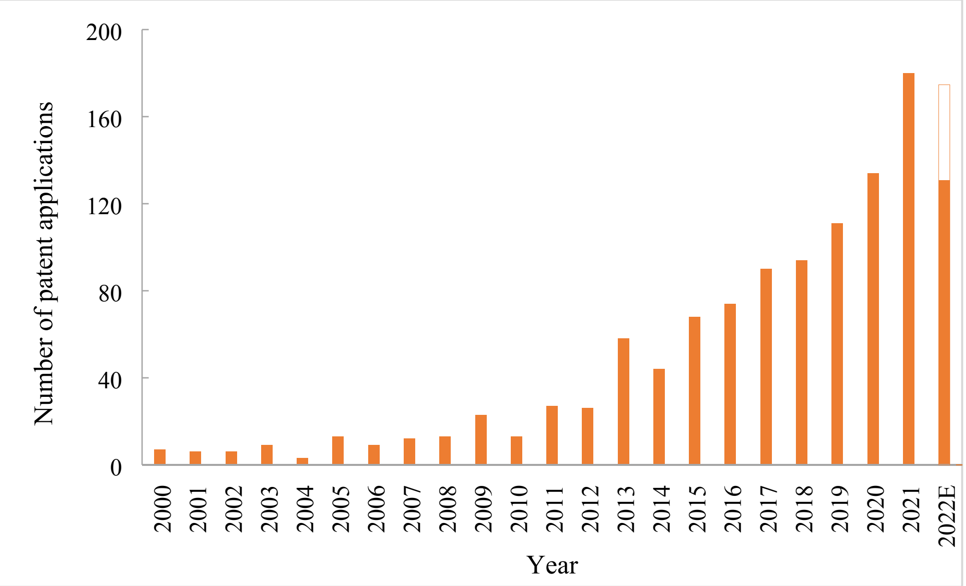

Figure S1. Global annual publications of patents related to mRNA therapeutics

SI-2. Trend over time for patents related to mRNA vaccines

The global patent application in the field of mRNA vaccines showed a trend of slow rise at first and then rapid rise (Figure S2). From 2000 to 2019, the number of related patents in this field was relatively small, with the annual number of patent applications within 50. After 2020, the number of related patent applications in this field increased rapidly from 55 in 2020 to 175 in 2021, and the number of patents in 2022 is expected to be more than 160.

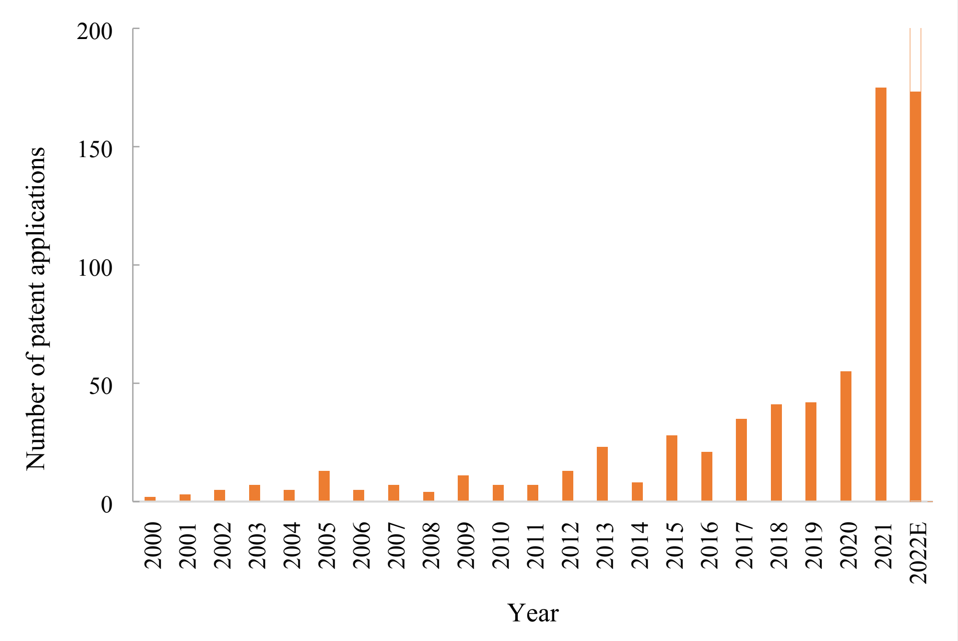

Figure S2. Global annual publications of patents related to mRNA vaccines

SI-3. Trend over timefor patents related to mRNA delivery systems

As shown in Figure S3, the annual number of patents in the field of mRNA delivery systems generally shows a rising trend, although the increase was more significant over the past several years. From 2000 to 2002, the number of patent applications in this field was small.

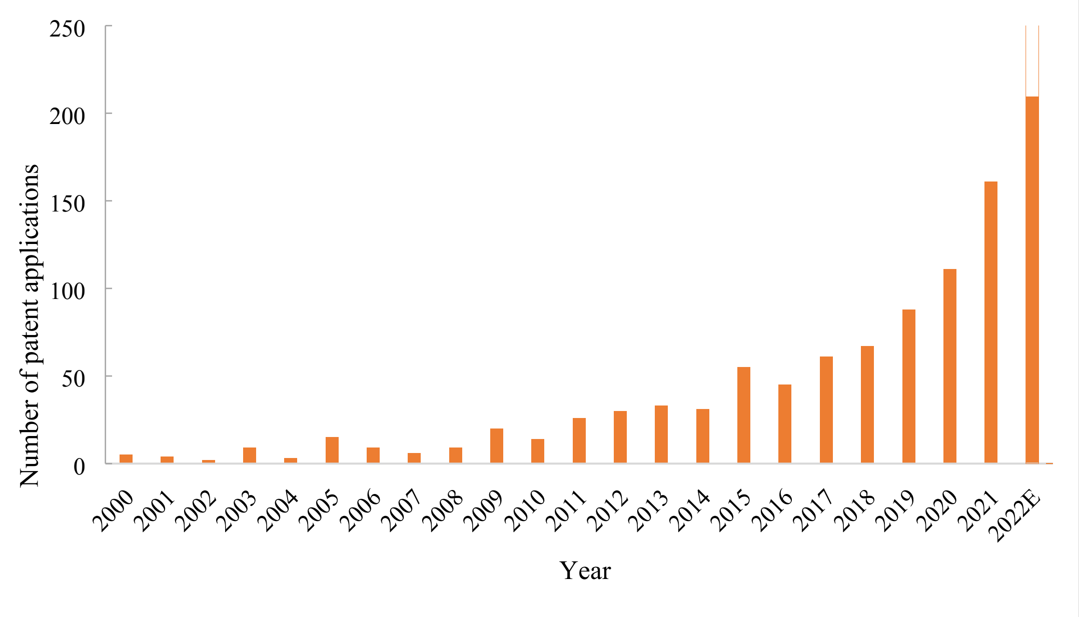

Figure S3. Global annual publications of patents related to mRNA delivery systems. Note: An estimated value (empty part) was provided for October through December of 2022 based the data collected up to the end of September (solid part).

SI-4. Trend over time for patents related to mRNA modification

From the annual trend, the number of global patent applications in the field of mRNA modification fluctuates, with a significant increase and peak in 2013, followed by a decline (Supplemental Figure S4). The trend of patent applications was relatively stable from 2015 to 2018, and after a brief decline in 2019, the number of patent applications showed a slow growth trend from 2020.

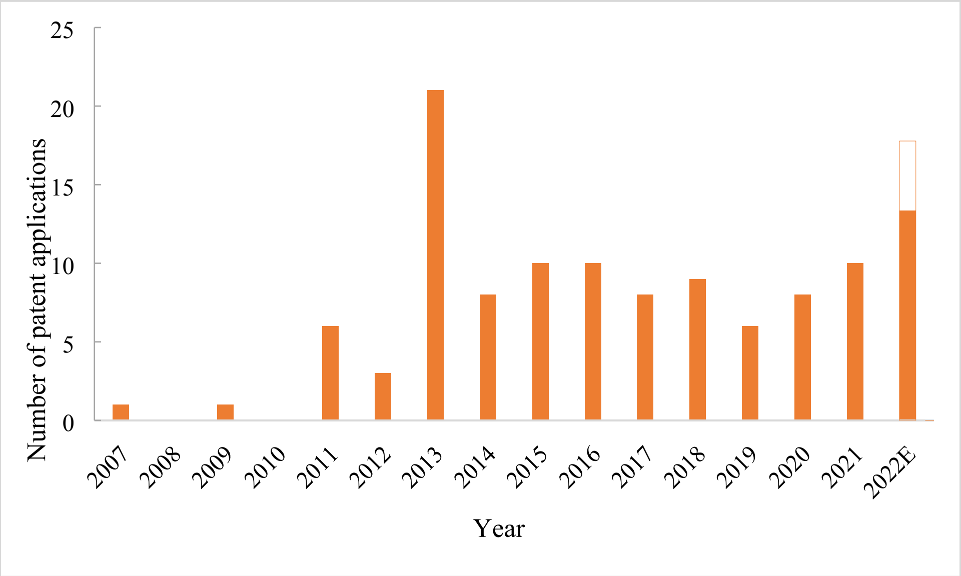

Figure S4. Global annual publication of patents related to mRNA modifications

SI-5. Analysis of research topics in mRNA therapeutics patents

From the distribution of patent research topics as indicated by cluster analysis of CAS index terms, the global effort on mRNA therapeutics focused on drug development methodologies, disease indications, and mechanism of actions such as signallng molecules, immune responses, etc.(Supplemental Figure S5). key concepts for drug development include antitumor agents, pharmaceutical carriers, pharmaceutical nanoparticles, pharmaceutical liposomes, etc (red dots). In the studies of diseases and their mechanisms (green dots), key concepts for disease indications and machnistic studies includeimmunoglobulins and their heavy chains, single-chain antibodies, cell proliferation, etc. Key concepts for immune studies (blue dots) include terms such as antigens, T cells, cancer immunotherapeutics, and chimeric antigen receptors. The signaling molecules (yellow dots) that have been highly explored include CD28 antigen, CD3 antigen, CD8 antigen, CD4 antigen, etc.

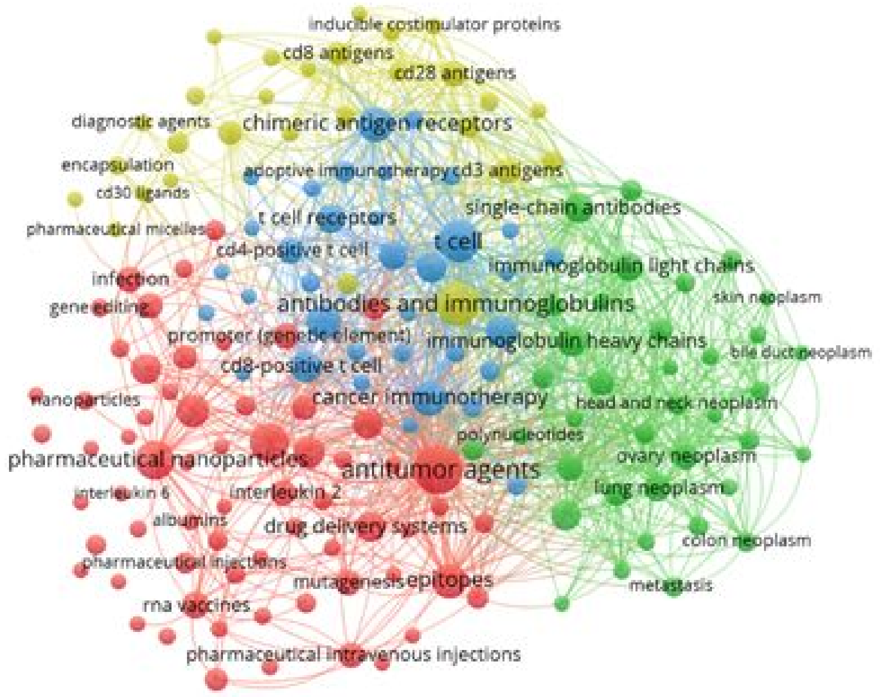

Figure S5. Distribution of topics in patents related to mRNA therapeutics

From the distribution of patent research topics, global research and development of mRNA-related delivery systems has mainly focused on delivery types, injection methods, drug carriers, and drug packaging (Figure S6). As can be seen in the Figure, although various drug delivery systems have been explored, lipid nanoparticles based on cationic lipids have been the major focus of most patents.

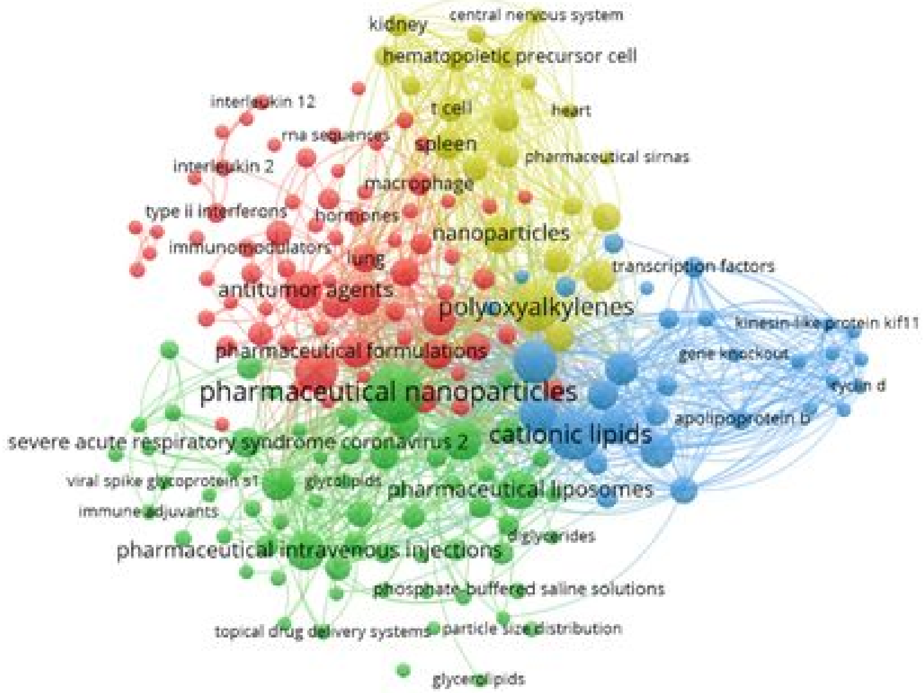

Figure S6. Distribution of topics in patents related to mRNA delivery systems

SI-6. Analysis of reserch topics in mRNA vaccine patents

From the distribution of patent research topics, the global mRNA vaccine field focuses on coronavirus vaccine research, tumor vaccine research, nucleic acid vaccine research, and other aspects (Figure S7). In the case of viral vaccines (red dots), key concepts include severe acute respiratory syndrome coronavirus 2, middle east respiratory syndrome related coronavirus), influenza vaccines, and others. Key concepts for cancer vaccines include lung neoplasm, mammary gland neoplasm, prostate gland neoplasm, indicating these diseases may be the focus of mRNA-based cancer vaccine development (green dots).

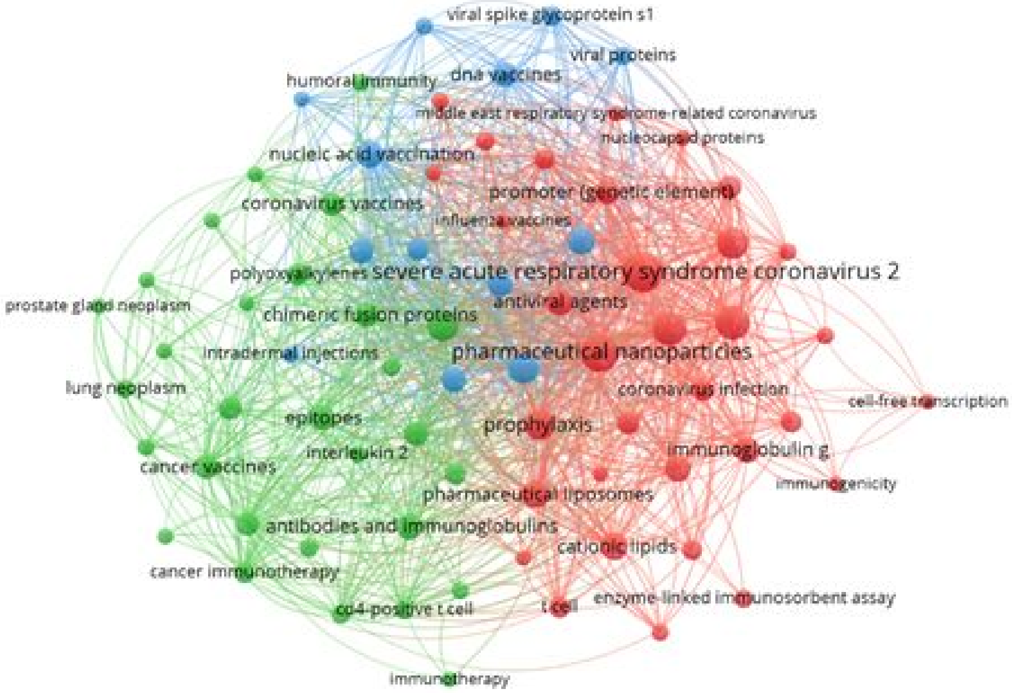

Figure S7. Distribution of topics in patents related to mRNA vaccines

SI-7. Analysis of research topics in patents related to mRNA modification

From the distribution of patent research topics, the global field of mRNA modification focuses on drug delivery types, carrier materials, nucleic acid modification, mechanism research, and other aspects (Supplemental Figure S8). Among them, in terms of the type of administration, key concepts include intraperitoneal injections, pharmaceutical intravenous injections, subcutaneous injections, etc. With regards to carrier materials, nanoparticles are infused with cationic lipids, pharmaceutical nanoparticles, pharmaceutical liposomes, etc. Nucleic acid modifications include oligonucleotide analogs, nucleotide analogs, peptide nucleic acids, etc. In terms of mechanism research, key concepts include transcription, growth factors, and membrane proteins, etc.

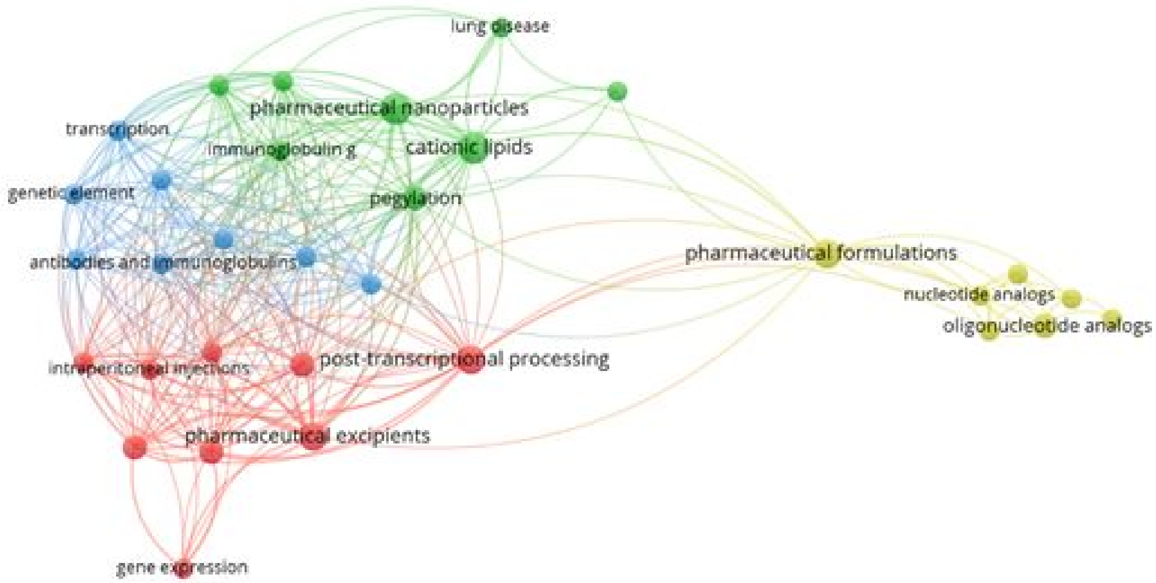

Figure S8. Distribution of topics in patents related to mRNA modification

SI-8. Top countries and organizations in the field of mRNA modification

Figure S9 and Table S1 show the countries and the organizations, respectively, with the most patents related to mRNA modifications.

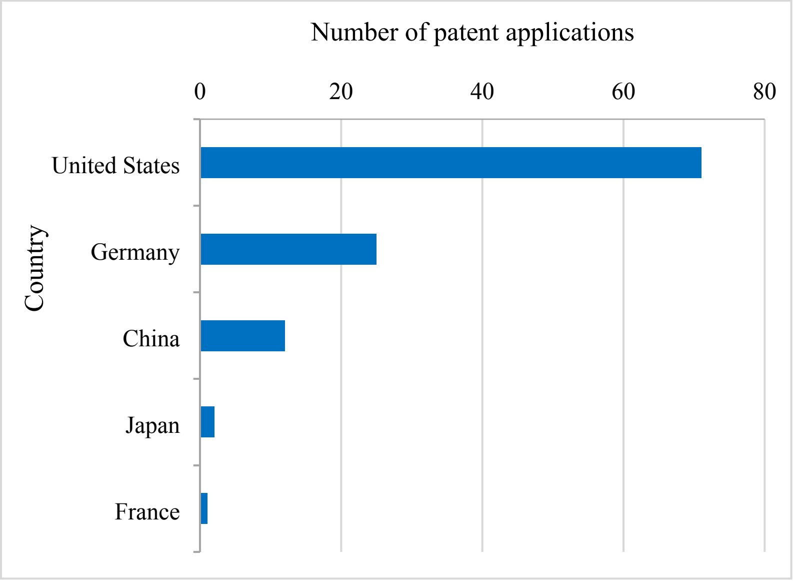

Figure S9. Top five countries with patents related to mRNA modifications

Table S1. Organizations with most patents related to mRNA modifications

Numbers	Organizations	Number of patent applications	Country	Organization type
**1**	ModernaTX, Inc	37	United States	Company

**2**	CureVac AG	12	Germany	Company
**3**	BioNTech AG	8	Germany	Company
**4**	Alnylam Pharmaceuticals, Inc	5	United States	Company
**4**	Translate Bio, Inc	5	United States	Company
**6**	Tron GmbH	4	Germany	Company
**6**	University of Pennsylvania	4	United States	University
**8**	Arcturus Therapeutics, Inc	3	United States	Company
**8**	Eberhard Karls Universitaet Tuebingen	3	Germany	University
**8**	Mount Sinai Hospital	3	United States	Scientific research institution
